# Structural Basis of Type 2A von Willebrand Disease Investigated by Molecular Dynamics Simulations and Experiments

**DOI:** 10.1371/journal.pone.0045207

**Published:** 2012-10-23

**Authors:** Gianluca Interlandi, Minhua Ling, An Yue Tu, Dominic W. Chung, Wendy E. Thomas

**Affiliations:** 1 Department of Bioengineering, University of Washington, Seattle, Washington, United States of America; 2 Department of Biochemistry, University of Washington, Seattle, Washington, United States of America; National Cerebral and Cardiovascular Center, Japan

## Abstract

The hemostatic function of von Willebrand factor is downregulated by the metalloprotease ADAMTS13, which cleaves at a unique site normally buried in the A2 domain. Exposure of the proteolytic site is induced in the wild-type by shear stress as von Willebrand factor circulates in blood. Mutations in the A2 domain, which increase its susceptibility to cleavage, cause type 2A von Willebrand disease. In this study, molecular dynamics simulations suggest that the A2 domain unfolds under tensile force progressively through a series of steps. The simulation results also indicated that three type 2A mutations in the C-terminal half of the A2 domain, L1657I, I1628T and E1638K, destabilize the native state fold of the protein. Furthermore, all three type 2A mutations lowered *in silico* the tensile force necessary to undock the C-terminal helix 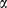
6 from the rest of the A2 domain, the first event in the unfolding pathway. The mutations F1520A, I1651A and A1661G were also predicted by simulations to destabilize the A2 domain and facilitate exposure of the cleavage site. Recombinant A2 domain proteins were expressed and cleavage assays were performed with the wild-type and single-point mutants. All three type 2A and two of the three predicted mutations exhibited increased rate of cleavage by ADAMTS13. These results confirm that destabilization of the helix 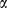
6 in the A2 domain facilitates exposure of the cleavage site and increases the rate of cleavage by ADAMTS13.

## Introduction

The multimeric and multi-domain protein von Willebrand factor (VWF) is essential to mediate adhesion of platelets to the site of vascular injury under high shear stress conditions like in arteries and arterioles [1], [2]. The A1, A2 and A3 domains of VWF have been recently studied intensively because of their critical role in the function of this protein. The A3 domain binds to the exposed subendothelium when a vessel injury has occurred, anchoring the VWF multimer. Then, the high shear generated by rapidly flowing blood activates VWF [3]. In particular, the A1 domain binds to platelet surface receptors glycoprotein Ib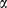 and this interaction has been shown to be strengthened by tensile force [4], [5]. A necessary element for proper physiologic function however is the secretion of so called ultralarge VWF multimers which are more active in binding to platelets than smaller VWF proteins [6]. This mechanism is counteracted by the metalloprotease ADAMTS13 which cleaves a scissile bond contained in the A2 domain of VWF [7], thus converting ultralarge VWF into smaller forms. ADAMTS13 is also a multi-domain protein and the interaction of its constituent domains with VWF is still an area of investigation [8]. Shear stress present in flowing blood is responsible for stretching the VWF protein and exposing the proteolytic site of the A2 domain such that ADAMTS13 can dock and cleave it. Taken together, shear stress is essential to activate VWF but at the same time it triggers its downregulation; this constitutes a very refined mechanism optimized to prevent the formation of blood clots where they are not needed.

This delicate blood coagulation mechanism can become out of balance when one of its constituent elements fails. For example, absence or malfunction of ADAMTS13 causes the disruption of the downregulation mechanism of VWF. This ultimately leads to pathologic thrombus formation and occlusion of atherosclerotic arteries which poses a life threatening risk [9]. On the other hand, mutations in the A2 domain are clinically known to cause excessive cleavage leading to the bleeding disorder called type 2A von Willebrand disease [10], [11]. The exact mechanism by which type 2A mutations alter the stability of the A2 domain and increase its susceptibility to ADAMTS13 cleavage has not been elucidated yet at the molecular level, although the structure and function of this protein have been investigated by numerous experimental studies [12].

**Figure 1:**
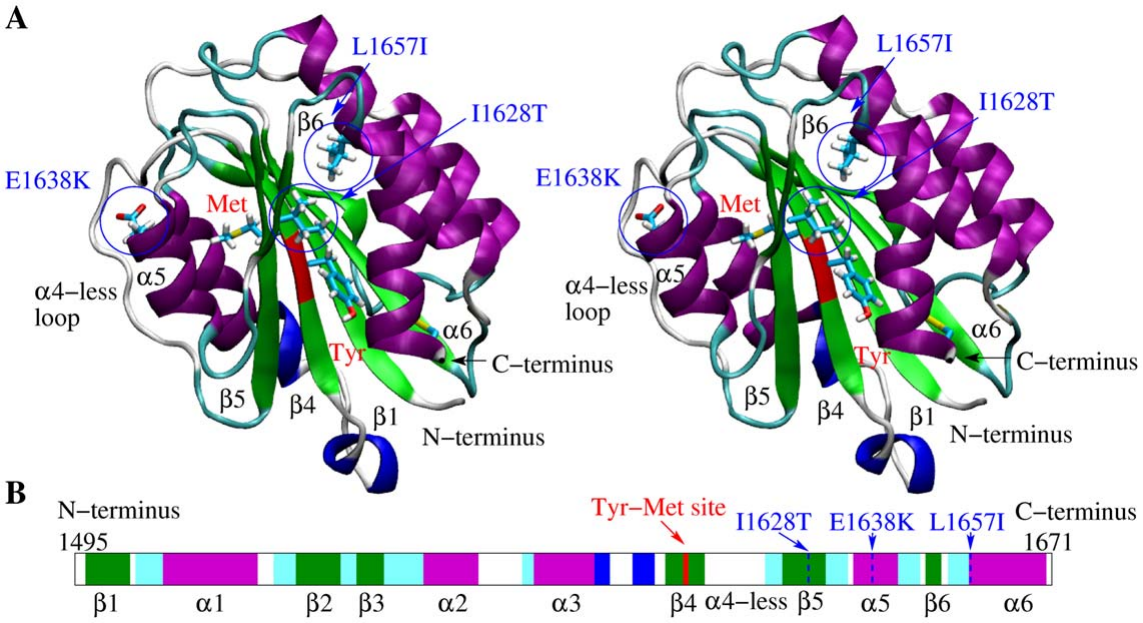
Tertiary and secondary structure of the A2 domain. (**A**) Stereoview of the A2 domain X-ray structure (PDB code 3GXB). The backbone of the proteolysis site is indicated in red and the rest of the protein is colored according to secondary structure elements with 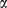 helices in purple, 3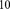 helices in blue, 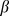 strands in green and turns in cyan. The side chains of Tyr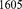 and Met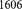, which are part of the cleavage site, and of the mutation sites Leu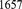, Ile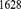 and Glu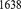 are drawn in the ball and stick representation. The three mutation sites are also indicated by blue circles. The labeling of 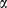 helices and 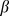 strands is the same as in reference [13]. (**B**) Secondary structure sequence. The cleavage site and the location of the three type 2A mutations investigated here are indicated. This figure was created with VMD [49].

The structure of the A2 domain solved through X-ray crystallography [13] presents a similar fold as the neighboring A1 and A3 domains, i.e., a central 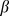 sheet consisting of six strands surrounded by mainly 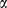 helices (Figure 1). However, the A2 domain presents only five instead of six 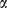 helices because a relatively long unstructured loop replaces the fourth helix (numbered from the N-terminus) at the analogous location in the folds of A1 and A3. This region is thus termed 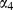-less loop [13]. In this manuscript, the same numbering for helices and strands is used as in a previous study [13] (Figure 1). Buried inside the protein is the proteolysis site (residues Tyr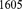-Met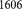) located in the 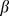
4 strand. Several single molecule force spectroscopy studies have shown that tensile forces exerted by rapidly flowing blood onto VWF are able to unfold the A2 domain. Furthermore, ADAMTS13 can cleave the A2 domain only if it is denatured [14]–[16]. However, no study has reported so far how mutations alter the mechanical regulation of the A2 domain thereby enhancing or decreasing its susceptibility to ADAMTS13. In particular, the absence of a disulfide bond linking the N-terminus of the protein with the C-terminal end of helix 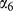 suggests that this region might be sensitive to tensile force. This disulfide bond is present in the homologous A1 and A3 domains and might be responsible for their higher thermodynamic stability [17] and mechanical resistance [16] compared to the A2 domain.

In this study, the effects of type 2A mutations located near the C-terminal helix of the protein were investigated by a combination of molecular dynamics simulations and cleavage experiments. Mutations were excluded which introduced an obvious disruption of the native state, i.e., mutating a hydrophobic residue into a charged side chain or vice versa. In total, three single point mutations linked to type 2A von Willebrand Disease, L1657I [18], I1628T [19], [20] and E1638K [21], [22], were selected for this study. Molecular dynamics analysis was used to characterize the effect of mutations onto the structural stability of the A2 domain with and without applied tensile force. The computational results were then validated against an ADAMTS13-induced cleavage assay using mutagenesis and protein engineering. A thorough structural understanding of the regulation of the A2 domain is essential to guide structure-based computational drug design [23] and discover novel therapeutic molecules to treat von Willebrand disease or thrombogenic illnesses.

## Results

### Analysis of the native state

In order to understand whether type 2A mutations alter the kinetic stability of the native state of the A2 domain, room temperature (300 K) simulations were run with the wild-type and the three single point mutants L1657I, I1628T and E1638K (Figure 1). All three mutations are clinically known to cause type 2A von Willebrand disease. In total, 12 simulations were run at 300 K, three for each mutant and the wild-type (Table 1).

**Table 1:** Simulation systems.

Name	Starting structure	Type 2A*^a^*	Tensile force*^b^*	Duration [ns]
WT_1,2,3*^c^*	Wild-type, PDB code 3GXB			3×40
L1657I_1,2,3*^c^*	L1657I	X		3×40
I1628T_1,2,3*^c^*	I1628T	X		3×40
E1638K_1,2,3*^c^*	E1638K	X		3×40
WT_pull_1,2,3*^c^*	WT_1 (10 ns), WT_2 (10+20 ns)*^d^*		X	3×55
L1657I_pull_1,2,3*^c^*	L1657I_1 (10 ns), L1657I_2 (10+20 ns)*^d^*	X	X	3×25
I1628T_pull_1,2,3*^c^*	I1628T_1 (10 ns), I1628T_2 (10+20 ns)*^d^*	X	X	3×25
E1638K_pull_1,2,3*^c^*	E1638K_1 (10 ns), E1638K_2 (10+20 ns)*^d^*	X	X	3×25
F1520A_1,2,3*^c^*	F1520A			3×40
I1651A_1,2,3*^c^*	I1651A			3×40
A1661G_1,2,3*^c^*	A1661G			3×40
F1520A_pull_1,2,3*^c^*	F1520A_1 (10 ns), F1520A_2 (10+20 ns)*^d^*	X	X	3×25
I1651A_pull_1,2,3*^c^*	I1651A_1 (10 ns), I1651A_2 (10+20 ns)*^d^*	X	X	3×25
A1661G_pull_1,2,3*^c^*	A1661G_1 (10 ns), A1661G_2 (10+20 ns)*^d^*	X	X	3×25

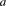
The mutation induces type 2A VWD. 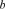
Tensile force was applied by pulling the C-terminus at constant velocity from the N-terminus. 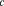
Three simulations were started for the wild-type and each mutant with and without applied tensile force; they are labeled with 1, 2 and 3, respectively. 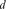
The pulling runs were started from snapshots sampled during the simulations with no tensile force, more specifically after 10 ns of run XXX_1 and after 10 and 20 ns of run XXX_2, where XXX is either WT or one of the mutations.

**Figure 2:**
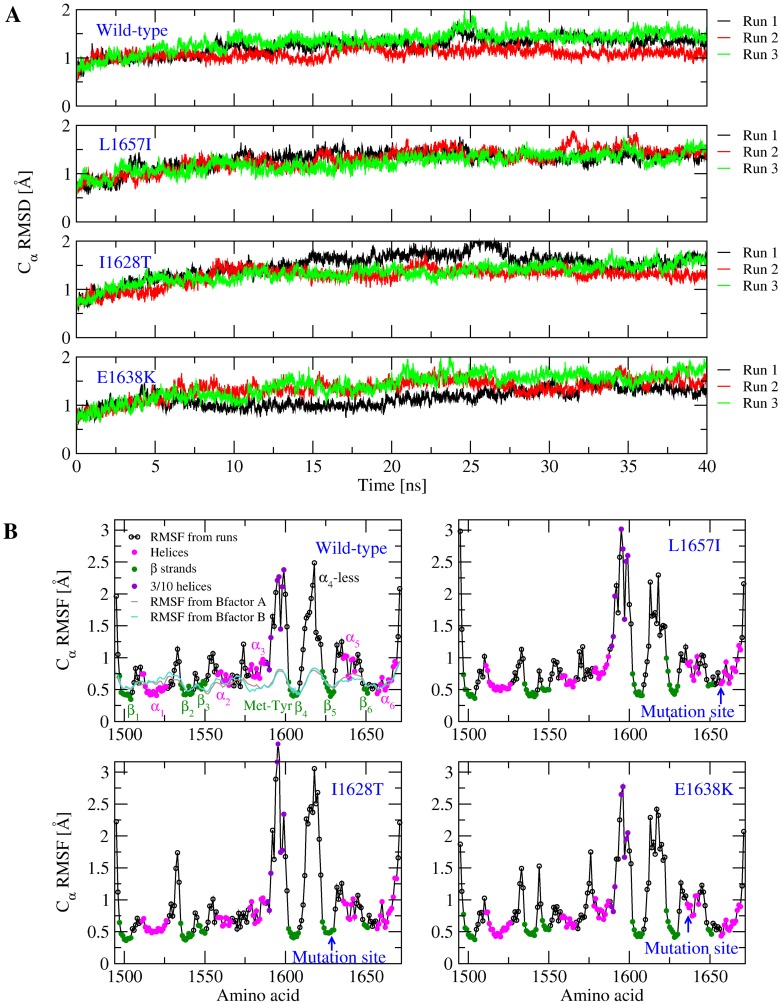
General analysis of the 300 K simulations. (**A**) Time series of the C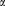 RMSD. (**B**) C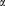 RMSF calculated from the 300-K simulations. The C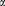 RMSF was calculated using the last 30 ns of in total 40 ns long 300 K simulations and averaged over three runs for each of the mutants and the wild-type. Solid magenta, green and violet circles indicate C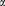 atoms contained in 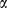 helices, 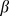 strands and 3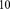 helices, respectively. Values of RMSF derived from crystallographic B factors of the C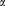 atoms (displayed in brown for chain A and turquoise for chain B together with the RMSF from the run with the wild-type) were calculated using the formula RMSF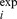 = 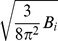, where 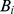 is the B factor of the C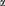 atom of residue 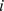 [50].

The simulations were first compared with the available crystallographic data in order to check for convergence. The C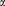 root mean square deviation (RMSD) from the initial conformation remained below 2 Å during the course of all simulations at 300 K with the wild-type and the mutants (Figure 2a). This indicates that the mutations did not significantly alter the overall fold of the A2 domain during the time course of the room temperature simulations. Also, the 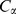 RMSD of helix 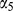 (which contains E1638K) and helix 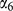 (near which the mutation sites L1657I and I1628T are located) remained generally below 1.5 Å in all simulations (Figure 1 and Figures S1 and S2). This is a further indication that secondary structure elements are likely conserved in the structure of the mutants. Most of the total C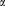 RMSD is probably accounted for by the motion of the 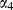-less loop and the 3/10 helix between 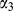 and 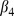. In fact, these regions fluctuate in the simulations more than the rest of the protein as indicated by the larger C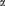 root mean square fluctuations (RMSF) in Figure 2b. This is in contrast with the crystallographic B-values according to which the 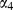-less loop and the 3/10 helix are much stiffer in the crystalline state than in the simulations (Figure 2b). This discrepancy is probably due to contacts between the two molecules found in the crystallographic unit and between neighboring cells. In general, there is qualitatively good agreement between the simulations and the crystallographic data concerning backbone flexibility (Figure 2b) validating that the runs sampled the conformational space of the folded state.

The trajectories were further analysed to estimate whether the three type 2A mutations cause a local destabilization of the A2 domain. Two types of analysis were performed for this purpose. First, statistical comparison of the 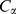 RMSF of the C-terminal helix, 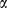
6, revealed the presence of larger fluctuations in the simulations with the L1657I and I1628T mutants compared with the wild-type simulations (Figure 3a). No statistically significant differences were observed with the E1638K mutant across the protein (data not shown). Second, the interaction energy between the mutated side chains and the rest of the protein or the rest of the simulated system (protein and bulk) was averaged over the sampled trajectories in order to detect local changes in enthalpy due to the mutations. For all of the mutants, there was an increase in the total interaction energy compared to the wild-type (Figure 3b) indicating that the mutations are enthalpically unfavorable. The side chains of residues Leu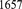 and Ile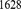 are located in a hydrophobic core which is shielded from the solvent by the C-terminal helix (Figure 1). Thus mutating these side chains is likely to cause a destabilization of the hydrophobic packing (Figure 3b). On the other hand, the mutation E1638K, located on the surface of helix 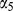 (Figure 1), introduces a positively charged side chain next to Arg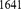, which is probably the reason for the higher coulombic energy (Figure 3b). It can be concluded that the mutations cause a local kinetic destabilization of the A2 domain native state, although the mechanism of destabilization might be different for E1638K than for L1657I or I1628T. The calculation of the interaction energy and of the 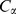 RMSF has been previously successfully used to identify stabilizing mutations in the C-terminal region of designed ankyrin repeat proteins [24], [25].

**Figure 3:**
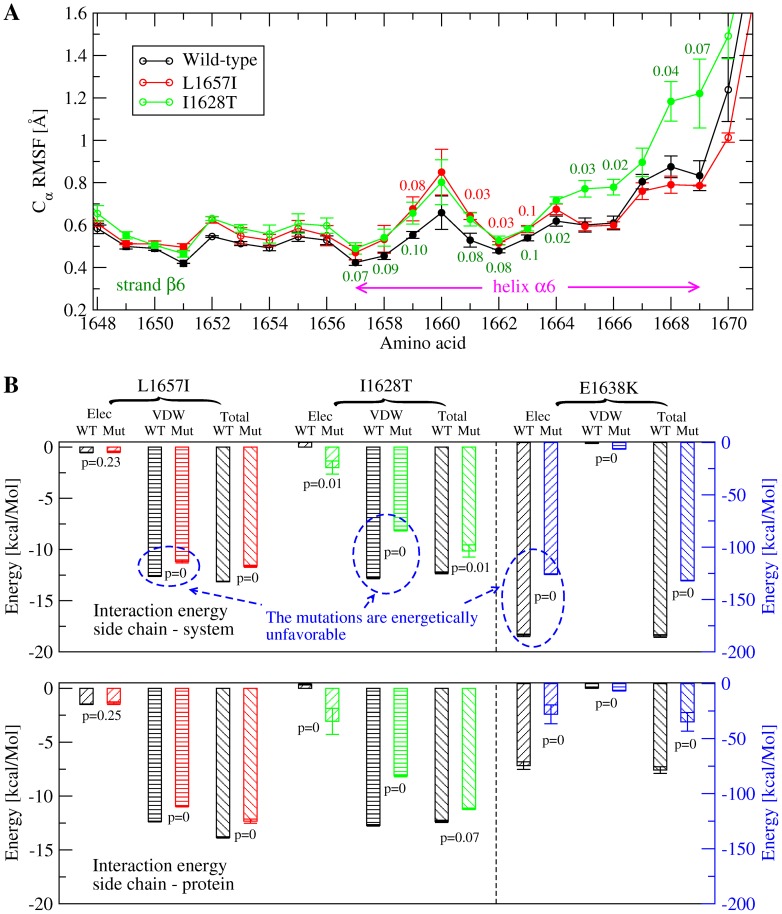
Effects of mutations onto the structural stability of the A2 domain. (**A**) C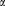 root mean square fluctuations of the C-terminal helix, 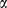
6, and strand 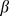
6. The values are calculated over the last 30 ns of in total 40-ns long room temperature simulations. Averages and standard error of the means are calculated over three independent runs for each mutant and the wild-type. The values reported next to the error bars are the correlation factors calculated from a single tailed student t-test. A difference is said to be statistically significant if the correlation factor is not larger than 0.05, whereas values between 0.05 and 0.10 are referred to as marginally statistically significant. Only correlation factors not larger than 0.10 are reported. (**B**) Interaction energy between a mutated side chain and (upper panel) the rest of the system (i.e., solvent and protein excluding the mutated side chain), or (lower panel) rest of the protein, subtracted from the interaction energy of the wild-type residue.

### Tensile force induced unfolding simulations

Starting from snapshots sampled during the 300 K runs, simulations were performed with an applied tensile force aimed at stretching the protein. The C-terminus of the A2 domain was pulled at constant velocity from the N-terminus whose coordinates were held fixed (see “Materials and Methods”). Three runs were started for each of the mutants and the wild-type, for in total 12 simulations (Table 1). The goal was to understand whether the mutations lower the force resistance of the A2 domain. The advantage of constant velocity versus constant force pulling simulations is that in the former the force ramps up until a breaking event occurs, after which the force usually drops. In this way, it is possible to monitor the presence of kinetic barriers in the force-induced unfolding pathway. Generally, if pulling is performed slowly enough, the direction of force is not relevant. Nonetheless, one simulation was performed where the N-terminus was pulled and a qualitatively similar sequence of events was observed as pulling from the C-terminus (Figures 4 and S3).

**Figure 4:**
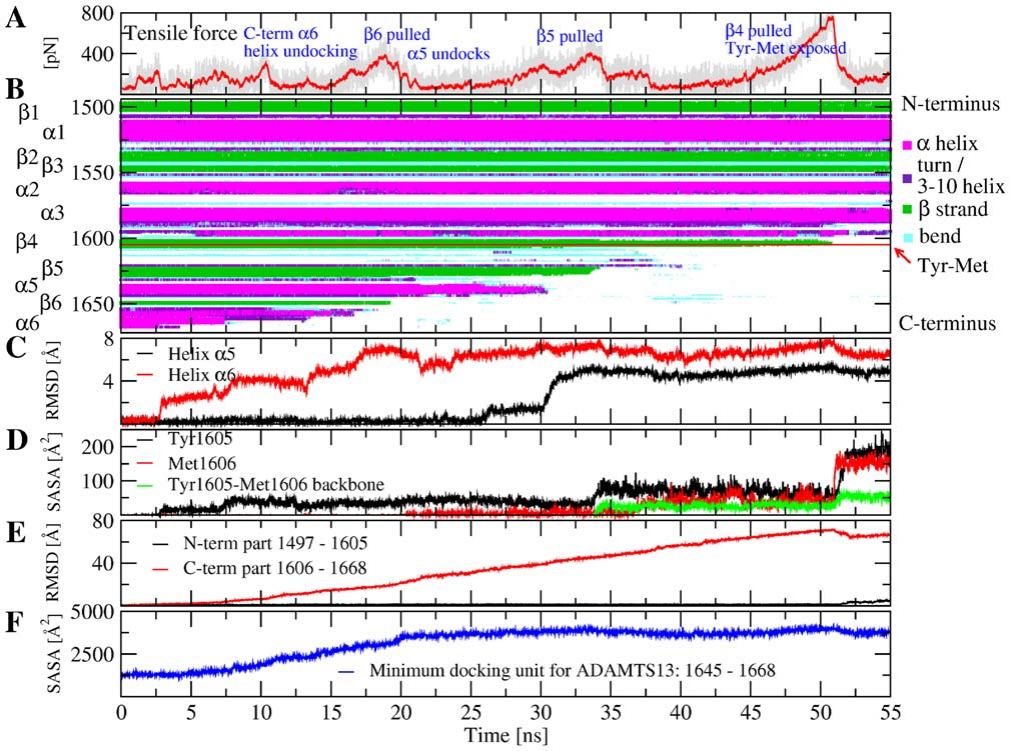
Time series of quantities measured during the pulling simulation WT_pull_1. Qualitatively similar unfolding pathways were observed in all runs with the wild-type and mutants (see Figures S4, S5, S6, S7, S8, S9, S10, S11, S12, S13, S14). (**A**) Applied tensile force. Events observed during the simulations corresponding to force peaks (i.e., sharp increases followed by drops) are indicated. (**B**) Formation of secondary structure elements. The colors are explained in the legend on the right. The position of the Tyr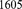-Met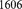 cleavage site is indicated by a red line and labeled on the right. (**C**) C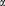 RMSD of the two C-terminus proximal helices 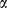
5 and 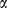
6. (**D**) Solvent accessible surface area of the Tyr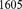-Met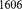 cleavage site. (**E**) C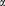 RMSD from the native state for the N-terminal part of the (residues 1497 to 1605) and the C-terminal part of the protein (residues 1606 to 1668). (**F**) Solvent accessible surface area of the minimum docking unit for ADAMTS13 (residues 1645–1668) identified in a previous experimental study [28].

#### General description of the unfolding pathway

**Figure 5:**
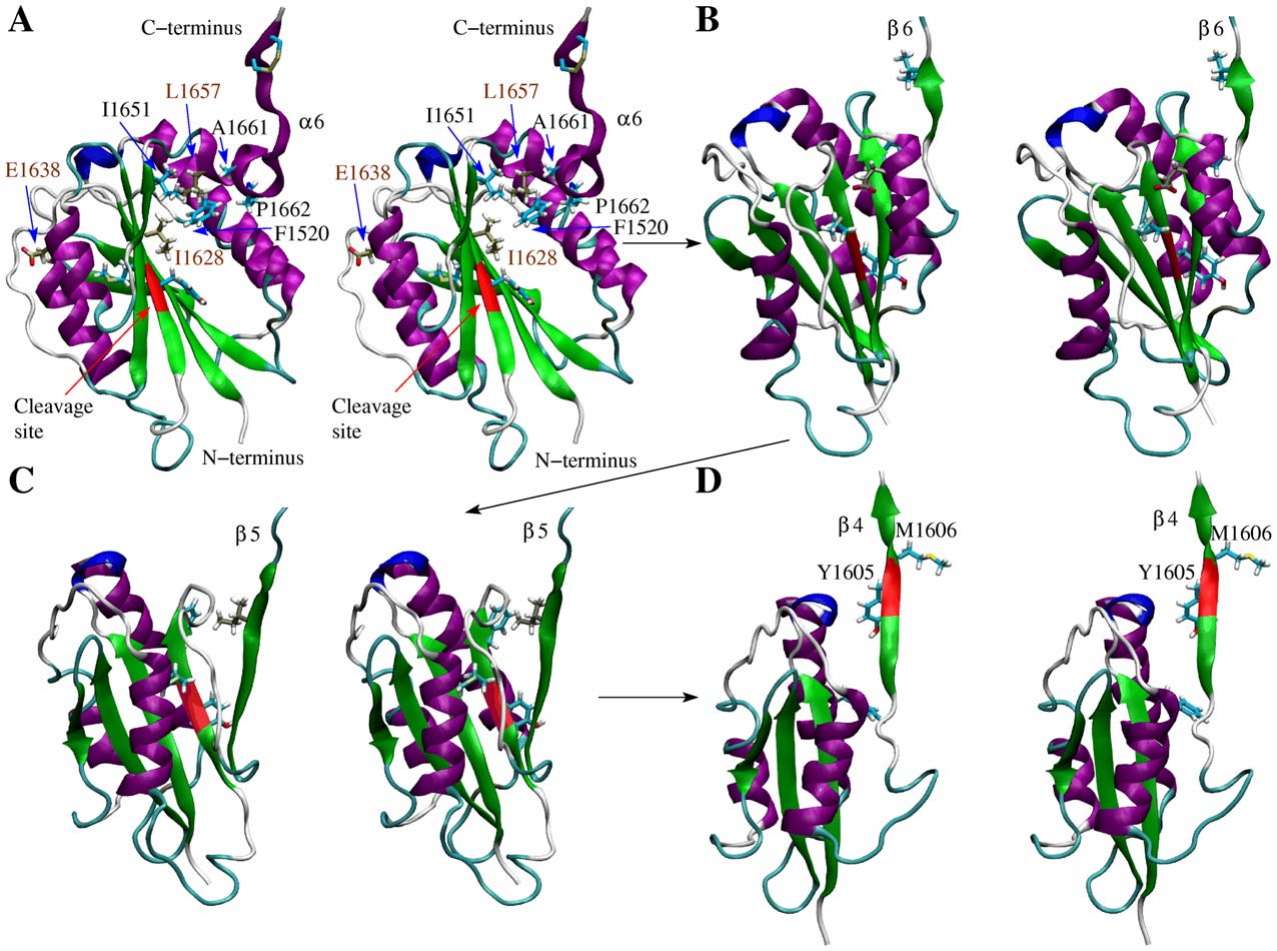
Snapshots along the unfolding pathway of run WT_pull_1 (stereoview). (**A**) After ca. 10.5 ns the N-terminal part of helix 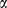
6 separated. The residues undergoing native side chain contacts with Ala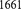 (see “Materials and Methods”) and the type 2A mutation sites are indicated in the ball and stick representation and labeled. The backbone of the Tyr-Met cleavage site is colored in red and its side chains are also displayed. Carbon atoms are colored in cyan, except those in the type 2A mutation sites which are colored in tan. (**B**) After ca. 20 ns strand 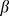
6 was pulled out and the side chain of Met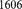 in the cleavage site was partially exposed. (**C**) After ca. 35 ns strand 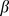
5 was pulled out and the hydroxyl group of Tyr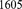 side chain became solvent accessible. (**D**) After ca. 51.5 ns strand 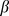
4 was pulled out and the cleavage site (side chains and backbone) was completely exposed to the solvent.

In general, the sequence of major events during unfolding was qualitatively similar between the simulations with the wild-type and mutants and was characterized by several rupture events which corresponded to peaks in the time series of the tensile force (Figure 4a). Secondary structure elements of the protein unfolded sequentially starting from the C-terminus leading to exposure of the proteolytic site (Figures 4b and 5, and movies S1 and S2). The first major force peak coincided with undocking of the second N-terminal turn of the C-terminal helix 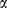
6 (Figures 4a, b and 5a). This was followed by undocking of strand 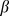
6 (Figure 5b), unfolding of helix 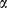
5 (Figure 4c), undocking of strand 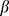
5 (Figure 5c) and finally pulling of strand 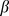
4 where the cleavage site is located (Figure 5d). This event caused full solvent exposure of the backbone of residues Tyr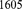 and Met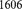 which is known to be cleaved by ADAMTS13 (Figure 4d). The simulation was stopped after this point, because unfolding of the rest of the protein is probably not necessary for the proteolytic process. Until rupture of 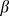
4, the N-terminal part of the protein (residues 1497 to 1605) remained in the same conformation as in the native state whereas the C-terminal part was unfolded (Figures 4b, e and 5d). The unfolding pathway presented here is qualitatively consistent with a previous MD study [26] performed with a model structure of the A2 domain [27] before the crystallographic structure was known.

Interestingly, the part of the protein comprising residues 1645 to 1668 (which includes strand 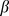
6 and the C-terminal helix 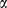
6) became solvent exposed while helix 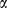
6 undocked from the rest of the protein and unfolded (Figures 4a, c and 5a). This region was identified in a previous experimental study to be the minimum docking unit of ADAMTS13 [28], and a subset of it (residues 1653 to 1668) was shown to be the recognition site for the spacer domain of ADAMTS13 [29]. Thus it can be speculated that unfolding of this region might facilitate docking of the proteolytic enzyme.

#### Effect of type 2A mutations on 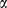
6 undocking

Two of the type 2A mutations investigated here, I1628T and L1657I contact residues in the C-terminal helix 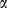
6 (Figure 1). Thus, in order to understand their effect onto the unfolding pathways, it was necessary to investigate whether they alter the force resistance of 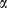
6 undocking, which is the very first event observed in all unfolding simulations with the wild-type and mutants (Figure 4a). Simulations performed with the mutant E1638K were also included in this analysis. Although the mutation site E1638K is located distally on 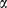
5, it is not excluded that destabilization of helix 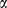
5 could propagate through the adjacent strand 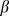
6 to the C-terminal helix.

**Figure 6:**
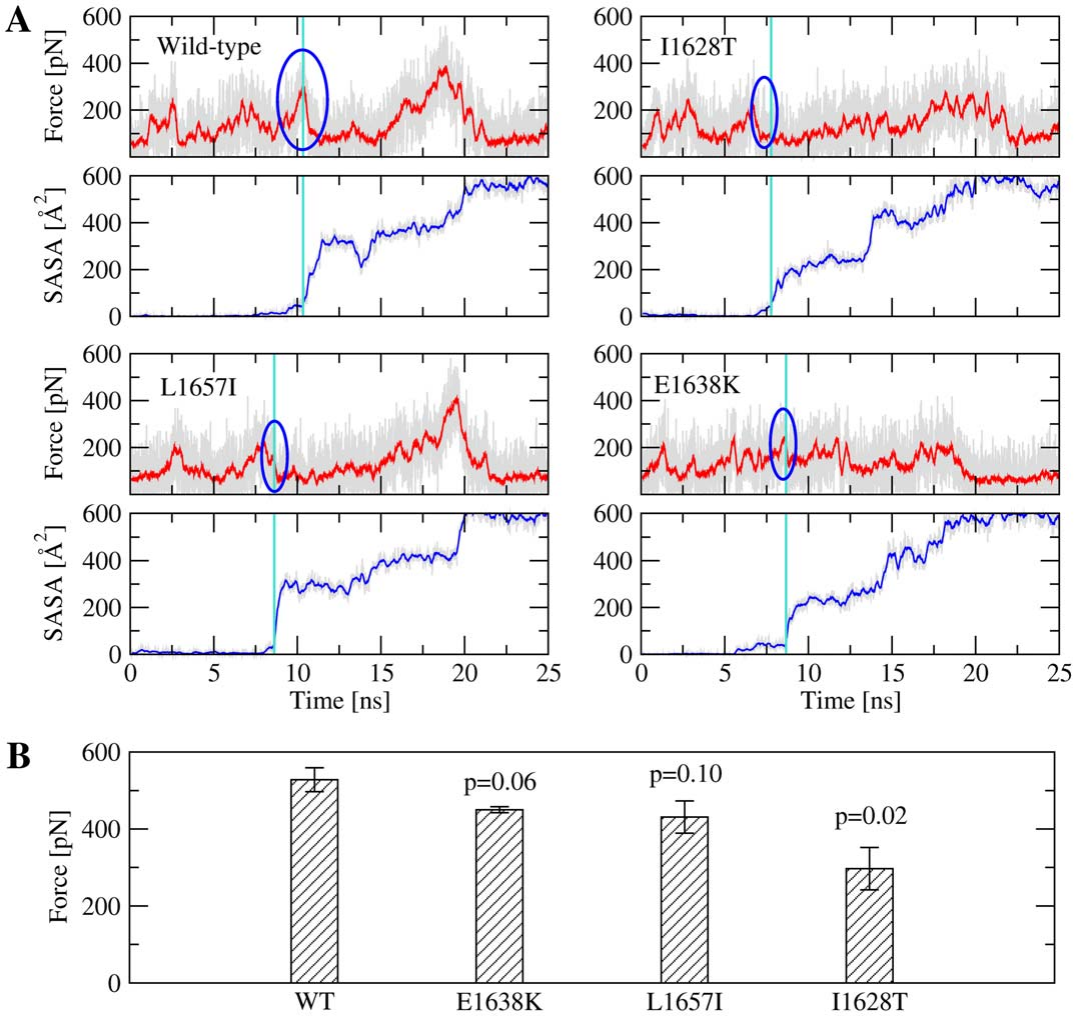
Analysis of key properties during pulling with the wild-type versus mutants. (**A**) Time series of the applied force and SASA of the C-terminal hydrophobic core. The force peak was determined by identifying the time point when the SASA exceeds 50 Å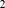 and searching for the highest value of the force within a 400 ps time window. 20-ps running average is indicated in red for the force and in blue for the SASA, respectively. (**B**) Mean and standard error of the force peaks corresponding to disruption of the C-terminal hydrophobic core averaged over three simulations (see also Figures S15 and S16). Reported are the correlation values (p-values) calculated from a single tailed student t-test.

Visual inspection of the trajectories with the wild-type revealed that the force peak observed during 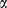
6 undocking coincides with separation of the side chain of Ala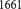 (located in the N-terminal half of helix 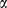
6) from the rest of the protein (Figure 5a). To better quantify this separation and how the mutations might affect it, residues were identified which formed native side chain contacts with Ala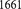 (see “Materials and Methods”), i.e., Phe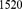, Ile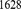, Ile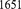, Leu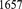 and Pro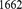. These amino acids together with Ala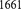 form a hydrophobic core which in the native state is buried by the C-terminal helix and is thus referred to as the “C-terminal hydrophobic core” for the purposes of this manuscript. In the case of the wild-type, solvent exposure of the C-terminal hydrophobic core (defined as the time point when the solvent accessible surface area exceeded 50 Å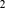) always coincided with a sharp drop of the tensile force (Figure 6a), indicating a major rupture event, i.e., separation of 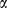
6 from the rest of the protein (Figure 5a). Strikingly, the force peak at which the C-terminal hydrophobic core became exposed was smaller in the case of the three mutants compared to the wild-type (Figure 6b-d). The smaller force peak is marginally statistically significant for E1638K and L1657I and statistically significant for I1628T (Figure 6e), indicating that these type 2A mutations are likely to lower the force resistance of the protein at least in the initial stages of unfolding.

These results are consistent with the energetic and RMSF analysis discussed in Section “Analysis of the native state”, which showed that the mutations cause a local destabilization of the native state of the A2 domain. Thus, a model can be suggested whereby the type 2A mutations investigated here destabilize the tertiary structure of the A2 domain, facilitating undocking of helix 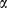
6 under tensile force or even shifting the thermodynamic equilibrium towards a state where helix 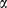
6 is undocked from the rest of the protein. This facilitates docking of the ADAMTS13 spacer domain and leads to further unfolding of the protein, making it susceptible to proteolysis through ADAMTS13.

### Validation of the 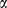
6 undocking mechanism

#### Further mutations near the C-terminal helix tested *in silico*

**Figure 7:**
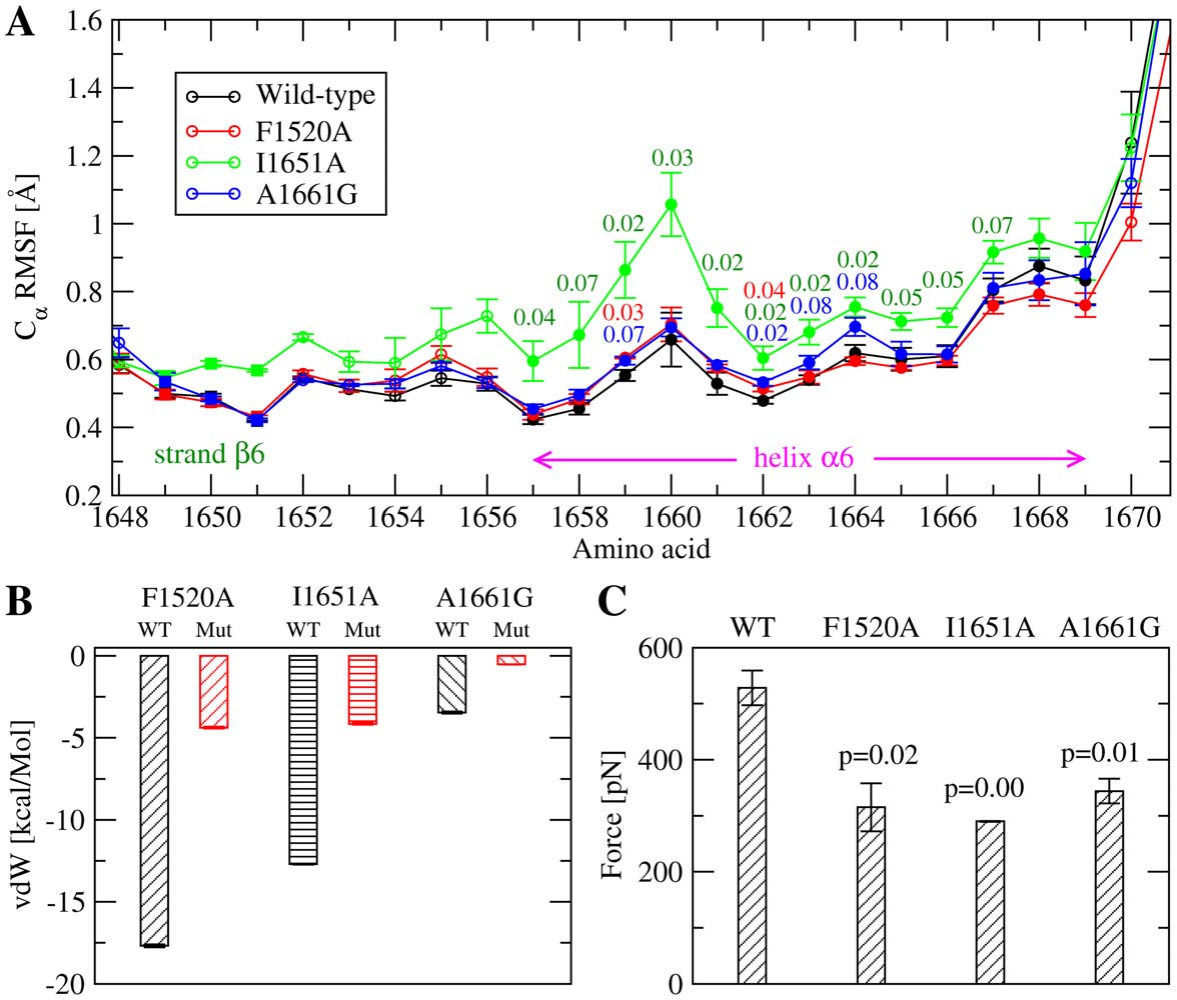
Analysis of mutations predicted to destabilize the A2 domain. (**A**) C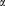 root mean square fluctuations of mutants compared to the wild-type. The values reported are averages over three runs. The bars indicate the standard error of the mean and the labels are the correlation p-values from a single-tailed student t-test (mutant versus wild-type). Reported are only p-values which are not larger than 0.1. (**B**) Van der Waals interaction energy between a side chain and the rest of the simulated system (i.e., rest of the protein and solvent). Compared are the energy of the wild-type residue (black) and the mutated side chain (red). The values are averages over three runs using the last 30 ns of in total 40 ns long runs at 300 K under static conditions (i.e., no force was applied). Electrostatic interaction energies are not shown because they are negligible compared to vdW. (**C**) Tensile force during exposure of the C-terminal hydrophobic core (see “Materials and Methods” for details about its measurement) in constant velocity pulling simulations with the mutants compared to the wild-type. The values are averaged over three runs and the standard error of the mean is indicated by error bars. The correlation p-values from the single tailed student t-test (mutant versus wild-type) are reported.

In order to validate computational predictions *in vitro* it is necessary to predict mutants that could exhibit the same phenotype as the clinically known pathological mutations. The goal here was to experimentally verify whether destabilization of the C-terminal helix of the A2 domain leads to an increased susceptibility to ADAMTS13. For this purpose, the three single-point mutants F1520A, I1651A and A1661G were analysed through MD simulations. These mutations are located in the C-terminal hydrophobic core near 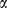
6 (Figure 5a) and are not known to cause type 2A von Willebrand disease. The same protocol was applied as for the type 2A mutations, whereby for each mutant three simulations were performed under static conditions (i.e., no tensile force was applied) and another three runs were done where the terminii were pulled from each other at constant velocity. Plots of the C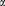 RMSF (Figure 7a) and calculation of the van der Waals (vdW) interaction energy (Figure 7b) suggested that the mutations are likely to destabilize the native state of the A2 domain. Also, the C-terminal helix of the single-point mutants undocked from the rest of the protein at a lower force than in the wild-type (Figure 7c). Thus, the designed mutations might induce a similar effect as type 2A mutations, i.e., destabilization of the A2 domain fold and subsequent increase in susceptibility to ADAMTS13 cleavage.

#### Cleavage experiments

**Figure 8:**
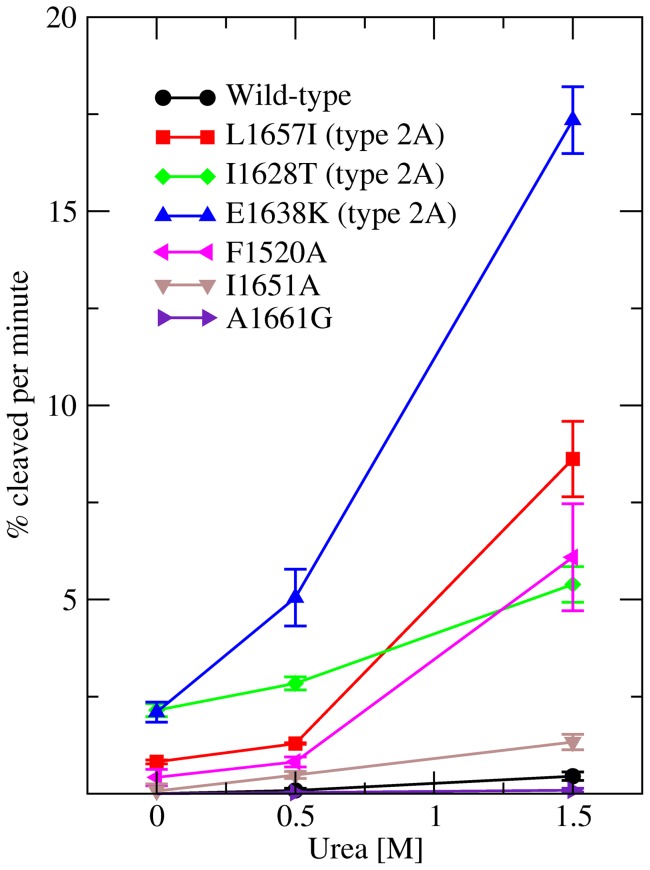
Cleavage rates of wild-type and mutant A2 fragments with ADAMTS13. Reported are the initial rates of cleavage expressed as the percent of A2 fragment cleaved per minute. The error bars indicate the standard deviation of three independent measurements.

A cleavage assay was used in order to verify that the structural destabilization through mutations predicted *in silico* leads to increased ADAMTS13 susceptibility. For this purpose, recombinant wild-type and mutant A2 domain proteins were expressed and the rate of ADAMTS13-induced cutting was measured in the absence and presence of urea as denaturant. As expected, the wild-type A2 domain was resistant to cleavage in the absence of denaturant (Figure 8). Of the six single-point mutants, five (the three type 2A mutations, F1520A and I1651A) showed susceptibility to ADAMTS13 cleavage in the absence of urea, although in various degrees (Figure 8). The mutants I1628T and E1638K were highly susceptible to cleavage by ADAMTS13 in the absence of urea, and were completely cleaved within 5 hours. On the other hand, L1657I, F1520A and I1651A showed moderate cleavage in the absence of denaturant. In general, the addition of urea increased susceptibility to the enzyme. Cleavage of the wild-type and mutant A1661G could be observed only in the presence of urea and both presented similar cutting rates within statistical significance and measuring accuracy (Figure 8).

It is interesting to note that the different amount of cutting for different mutants might be related to the physico-chemical properties of a particular mutation. The mutants E1638K and I1628T presented the highest rate of cleavage, probably because E1638K causes a strong electrostatic repulsion with a vicinal arginine, whereas I1628T introduces a hydrophilic side chain in a hydrophobic core. In contrast, the mutants L1657I, F1520A and I1651A have a relatively low rate of cleavage in the absence of urea probably because they involve more conservative mutations as they preserve hydrophobic side chains. Urea accelerates the rate of unfolding and thus also the rate of cleavage. However, a higher urea concentration might change the relative cutting rates between the various mutants. For example, the mutant I1628T is cleaved more easily than the mutants L1657I or F1520A in the absence of denaturant but no statistically significant difference is observed in the presence of 1.5 M urea (Figure 8).

## Discussion

Through a combination of MD simulations and a cleavage assay, the present study investigated the unfolding pathway of the A2 domain under tensile force and how it is affected by mutations that increase susceptibility to ADAMTS13 cleavage. Pulling simulations with the wild-type indicated that under tensile force the C-terminal part of the A2 domain unfolds exposing the cleavage site while the N-terminal part retains its native state conformation (Figure 5). Thus only type 2A mutations were selected that are located in the C-terminal part and do not introduce an obvious structural destabilization. The investigated mutations, E1638K, I1628T and L1657I, were found to kinetically destabilize the native state fold of the A2 domain (Figure 3) and to lower the force necessary to undock the C-terminal helix 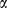
6 from the rest of the protein (Figure 6). Undocking of 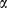
6 is also the very first event observed during unfolding (Figure 5) and this region is known to be the recognition site for the spacer domain of ADAMTS13 [30]. Thus these results suggest that destabilization of helix 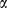
6 might induce a higher susceptibility to cleavage. In order to test this, three mutations not known to cause type 2A von Willebrand Disease, F1520A, I1651A and A1661G, were also predicted *in silico* to destabilize helix 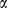
6. All six single-point mutants and the wild-type were then tested in a ADAMTS13-induced cleavage assay. The experiments showed that while the wild-type is resistant against cutting unless 1.5 M urea were added, all mutants with the exception of A1661G were cleaved in the total absence of urea.

The findings reported here for the type 2A mutations are in better agreement with the clinical phenotype than a previous study which used the entire VWF protein instead of just the A2 domain and found that I1628T does not increase cleavage by ADAMTS13 [31]. This indicates that the assay used here is more sensitive to the effects of mutations than the previous study. However, it would be interesting to test through single molecule force spectroscopy experiments, for example using optical [14] or magnetic tweezers [31], whether the mutation A1661G decreases the force resistance of the A2 domain as predicted *in silico*. This would help clarify the discrepancy between the simulations and the cleavage assay concerning A1661G. This discrepancy might be related to the binding specificity of ADAMTS13, which binds to the sequence between residues 1645 and 1668 of the A2 domain, encompassing Ala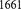 [28]. In fact, previous cleavage experiments with peptide substrates, consisting of the 78 C-terminal amino acids of the A2 domain, showed that mutations in the sequence between residues 1653 and 1663 reduced the rate of cutting, whereas the wild-type 78 amino acid sequence was readily cleaved [28]. This suggests that the mutation A1661G might destabilize the A2 domain fold as predicted *in silico*, but at the same time it might abolish recognition by the enzyme, such that cleavage is not observed.

The results from the present study suggest a mechanistic model for the interaction between the A2 domain and the enzyme ADATMS13. Studies based on binding assays and mutagenesis have determined that docking of ADATMS13 domains onto specific segments of A2 is essential for the proteolytic function [32]. Thus, unfolding of the A2 domain is necessary not only to expose the cryptic site but also for proper recognition by ADAMTS13. The first major event during unfolding is undocking of helix 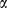
6 which is shown here to be facilitated by disruptive mutations located in its vicinity. Besides exposing part of the hydrophobic core and facilitating further unfolding, undocking of 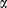
6 allows binding of the ADAMTS13 spacer domain [29]. In fact, specific residues in the spacer domain have been recognized to interact with a C-terminal segment of A2 that includes 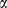
6 and this interaction has been determined to be essential for the isolated A2 domain to be efficiently cleaved [33], [34]. Unfolding then proceeds through several intermediates as further 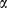 helices and 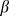 strands are pulled one after the other from the native fold (Figure 4). The presence of intermediate states is likely to reduce floppiness of the unfolding A2 domain backbone allowing further domains of ADATMS13 to dock at a relatively lower entropic cost. For example, the disintegrin-like domain of ADAMTS13 has been shown to interact with Asp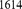 in the 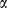
4-less loop of A2 thus correctly orienting the scissile bond towards the metalloprotease domain [35]. It needs to be mentioned that additional binding sites are available to ADAMTS13 in full length VWF because disruptive mutations in the spacer domain have been shown to alter the cleavage rates of full length VWF in a less dramatic way than the isolated A2 domain [33]. The VWF D4 domain for example is known to bind to regions of ADTAMS13 outside of the spacer domain [36]. Nonetheless, the interaction between the spacer domain and A2 contributes to the proteolytic mechanism even if it is not essential in full length VWF.

The C-terminal coil of helix 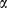
6 presents two adjacent cysteine residues, Cys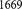 and Cys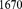, that form a buried disulfide bond. It has been suggested that this vicinal disulfide bond acts as a “molecular plug” stabilizing helix 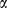
6 [13], [32]. A mutagenesis study showed that removing this disulfide bond increases the susceptibility of the A2 domain towards proteolysis through ADATMS13 [37]. This is in agreement with the mechanism presented here, whereby destabilization of helix 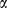
6 triggers unfolding of the A2 domain and docking of the proteolytic enzyme.

Crystallographic structures of the A2 domain have been recently reported that show the presence of a calcium ion in the vicinity of the cleavage site [15], [38]. Although there is consensus in the literature that calcium reduces cleavage of the A2 domain through ADAMTS13 [15], [38], [39], there is conflicting evidence whether it alters the unfolding pathway of the A2 domain. A previous molecular dynamics study [38] and single molecule force spectroscopy experiments [15] suggested that a calcium ion bound to the A2 domain introduces an intermediate in the unfolding pathway under tensile force. In contrast, a recent single molecule force spectroscopy study showed that calcium influences only the refolding pathway of the A2 domain [39]. The simulations presented here were performed with a crystallographic structure which does not contain any bound calcium ion. However, the binding site of the calcium ion is located distally from the C-terminus and thus it is not likely to influence the destabilization mechanism of the C-terminal helix observed here. Furthermore, the simulations results are in agreement with the cleavage experiments despite these were performed in the presence of 2 mM of calcium. Thus it can be speculated that calcium might not significantly affect the unfolding pathway of the A2 domain, but as pointed out by a recent study [39] it might reduce the amount of time when the scissile bond is available to ADATMS13 for cleavage.

Understanding how different mutations affect the stability of the A2 domain might be relevant also to protein engineering. For example, the A2 domain could be used as a scaffold in biotechnological applications where force sensitivity is required. In this context, it would be interesting to also engineer mutants that make the A2 domain more resistant to cleavage by increasing the stability of the C-terminal helix. For example, mutating P1662 in 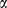
6 to an alanine or leucine should increase 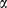 helical propensity and improve packing of the hydrophobic core. A previous study showed that stabilizing the C-terminal helices of designed ankyrin repeat proteins dramatically increased the thermodynamic stability of the native state and eliminated an intermediate state in equilibrium refolding experiments [24], [25]. These two studies together indicate that local stabilization or destabilization of a protein through mutations can lead to an increase or decrease, respectively in thermodynamic stability. This is of interest in particular to protein engineers and it highlights the usefulness of MD simulations, when fruitfully combined with experiments, in determining the weaker links in a scaffold.

The present work is the first study which attempts to explain the increased susceptibility of the A2 domain to ADAMTS13 cleavage caused by type 2A mutations based on the three-dimensional structure. This can be helpful to structure based drug design. For example, a drug targeted at patients suffering of type 2A or acquired von Willebrand disease should be designed in a way that it stabilizes the C-terminal helix of the A2 domain. Conversely, an anti-thrombotic drug molecule should be able to insert itself into a groove between the C-terminal helix and the core of the protein in order to favor the undocked conformation of the helix making the protein more susceptible to proteolysis. Current anti-thrombotic drugs have the disadvantage that they need to be administered in large doses to be effective and a patient needs to be taken off the drugs a few days before a surgery to prevent excessive bleeding [40]. Better anti-thrombotic therapeutics are wishful which are more efficient and can be turned off much more quickly when necessary. Advances in the understanding how VWF works at atomic level of detail is fundamental to guide structure based drug design.

## Materials and Methods

### Simulations

#### Initial conformations

The simulations with the wild-type were started from the crystallographic structure with PDB code 3GXB [13]. Mutants were constructed per homology by replacing the corresponding side chain in the wild-type and subsequently performing with the program CHARMM [41] 100 steps of steepest descent minimization in vacuo while the positions of all atoms except the mutated residue were kept fixed. The online VWF database of the University of Sheffield (http://vwf.group.shef.ac.uk) was used to search for type 2A von Willebrand disease mutations.

#### General setup of the systems

The MD simulations were performed with the program NAMD [42] using the CHARMM all-hydrogen force field (PARAM22) [43] and the TIP3P model of water. The different simulations systems are summarized in Table 1. The proteins were inserted into a cubic water box with side length of 80 Å, resulting in a system with in total ca. 50,000 atoms. In the simulations where a tensile force was applied, a rectangular water box was used as described in detail below. Chloride and sodium ions were added to neutralize the system and approximate a salt concentration of 150 mM. The water molecules overlapping with the protein or the ions were removed if the distance between the water oxygen and any atom of the protein or any ion was smaller than 3.1 Å. To avoid finite size effects, periodic boundary conditions were applied. After solvation, the system underwent 500 steps of minimization while the coordinates of the heavy atoms of the protein were held fixed and subsequent 500 steps with no restraints. Each simulation was started with different initial random velocities to ensure that different trajectories were sampled whenever the same primary sequence was simulated. Electrostatic interactions were calculated within a cutoff of 12 Å, while long-range electrostatic effects were taken into account by the Particle Mesh Ewald summation method [44]. Van der Waals interactions were treated with the use of a switch function starting at 10 Å and turning off at 12 Å. The dynamics were integrated with a time step of 2 fs. The covalent bonds involving hydrogens were rigidly constrained by means of the SHAKE algorithm with a tolerance of 10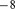. Snapshots were saved every 10 ps for trajectory analysis.

#### Equilibration and runs with no tensile force

Before production runs, harmonic constraints were applied to the positions of all heavy atoms of the protein to equilibrate the system at 300 K during a time length of 0.2 ns. For those systems where mutations were introduced per homology, harmonic constraints were kept on all heavy atoms except those of the mutated residue and the neighboring amino acids, and equilibration was continued for another 2 ns. After this equilibration phase, the harmonic constraints were released. The runs performed under static conditions, i.e., with no external force applied lasted in total 40 ns, and the first 10 ns of unconstrained simulation time were also considered part of the equilibration and were thus not used for the analysis. During the equilibration and in all runs with no tensile force, the temperature was kept constant at 300 K by using the Langevin thermostat [45] with a damping coefficient of 1 ps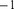, while the pressure was held constant at 1 atm by applying a pressure piston [46].

#### Constant velocity pulling

The simulations with applied tensile force were started from snapshots sampled during the runs with no tensile force described above (see also Table 1). The protein and a bulk layer of 6 Å were removed from the cubic water box and placed into a rectangular water box of 160 Å in the direction of pull and 80 Å in the other two directions. The system was then equilibrated at 300 K as described above. Positional restraints were then applied to the coordinates of the C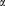 atom of the N-terminus. The C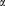 atom of the C-terminus was attached through a virtual spring with a stiffness constant of 2 kcal/mol to a dummy atom that was pulled at a constant velocity of 5 Å/ns. The initial direction of pull was parallel to the axis through the fixed N-terminal C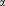 atom and the pulled atom. As the dummy atom is pulled, the spring extends. Using Hook's law, the resulting applied tensile force is defined as 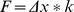, where 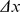 is the extension and 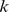 the stiffness constant of the spring. The tensile force can then be plotted in function of time in order to monitor rupture events. It needs to be noted that from a physical point of view the direction of force is not relevant, because the protein would rotate as a rigid body until the axis through the pulled atoms is aligned parallel to the direction of pull, if this was not the case at the start of the simulation. Similarly, it is generally assumed that if the pulling is performed gently enough the force will propagate through the protein and it will not matter which atom is fixed and which one is pulled. Each pulling simulation was run for 25 ns when the protein extension had reached a length within 20 Å from the longest dimension of the water box. In the case of the wild-type the simulation was continued after placing the protein in a water box with dimensions of 250×65×65 Å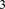 for 15 ns and subsequently into another box of 300×65×65 Å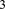 side lengths for another 15 ns, totaling 55 ns of pulling. The pulling simulations with the mutants were not extended beyond 25 ns because the unfolding pathways were qualitatively similar to the wild-type and the salient events happened during this first phase.

#### Native side chain contacts

In order to determine the side chains contained in the hydrophobic packing buried by the N-terminal part of the C-terminal helix 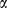
6 (named here C-terminal hydrophobic core), the native side chain contacts of Ala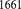 were determined. A side chain contact is defined to occur when the distance between the centers of mass of two side chains is not larger than 6 Å. Contacts present in a least 60% of the simulation frames of at least one simulation with no tensile force are defined as native.

### Experiments

#### Construction of expression plasmids for VWF A2 wild-type and mutants

DNA sequence encoding the wild-type VWF A2 domain (Asp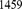-Gly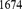) was amplified by PCR with primers that included a BamHI site at the 5′ end and a NotI site at the 3′ end of the A2 domain. The amplified fragment was cloned by TA-cloning into the vector pCR2.1-TOPO (Invitrogen, Carlsbad, CA). Mutations in the VWF A2 domain were introduced into the wild-type fragment according to the protocol of the QuikChange site-directed mutagenesis kit (Stratagene, La Jolla, CA). The sequence of the wild-type and mutant VWF A2 fragments were confirmed by DNA sequencing. Inserts containing the wild-type and mutant VWF A2 fragments were excised from the pCR2.1-TOPO vector by BamHI and NotI digestion and inserted into the corresponding sites in the pNBiosecPC4 expression vector [47]. In this expression system, recombinant proteins were secreted with dual tags: an N-terminal biotin-tag and a C-terminal protein C epitope tag [47].

#### Expression of recombinant VWF A2 fragment

Expression plasmids were transfected into HEK293 Tet-On cells with lipofectamine 2000 (Invitrogen, Carlsbad, CA). Transient expression was induced by doxycycline (2 mg/ml) in the presence of biotin (50 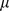
M) in FreeStyle 293 serum-free culture medium (Invitrogen, Carlsbad, CA) for 72 hr as previously described [48]. Culture medium containing secreted recombinant VWF A2 fragments was desalted over Sephadex G25 (GE Healthcare, Piscataway, NJ) into 10 mM Hepes pH 7.4, 2 mM CaCl2 to remove unincorporated biotin. A mixture of protease inhibitors (1% v/v, Protease Inhibitor Cocktail, Sigma, St. Louis, MO) was added to the recombinant fragments and stored at −80° C before use.

#### Expression of recombinant ADAMTS13

Recombinant ADAMTS13 was expressed with a C-terminal biotin tag with the pCBioSec vector in stably transfected HEK293 Tet-On cells as previously described [48]. Serum-free FreeStyle 293 medium (Life Technologies Corp) containing recombinant ADAMTS13 was concentrated tenfold by centrifugation in an Ultracel 10K centrifugal filter (Millipore, Billerica, MA) and desalted over Sephadex G-25 to remove biotin and low molecular weight molecules. The concentrated recombinant ADAMTS13 preparation was treated with a mixture of protease inhibitors (Protease Inhibitor Cocktail, 1% v/v, Sigma, St. Louis, MO) and stored at −80°C. Recombinant ADAMTS13 was quantified in western blots probed with streptavidin-HRP by comparison to serial dilutions of a reference preparation of biotinylated albumin, which contains 4 moles of biotin per mole of albumin, prepared by the chemical biotinylating agent ChromaLink (SoluLink, San Diego, CA). The extent of biotinylation with ChromaLink was determined by absorption spectroscopy of a chromophore in the biotin linker. Recombinant ADAMTS13 was used without further purification.

#### ADAMTS13 cleavage assays

The rates of cleavage of recombinant A2 fragments were measured by incubating 20 ng of each recombinant A2 fragment with 4 ng of recombinant ADAMTS13 in 10 mM Hepes, pH 7.2, 2 mM CaCl2, with or without urea, at 37°C for varying amount of time. For A2 fragments that were cleaved slowly, cleavage reactions were stopped by EDTA (final 10 mM) at 0, 10, 20, 30, 60 and 120 min. For A2 fragments that were cleaved rapidly, cleavage reactions were stopped by EDTA at 0, 5, 10, 20 and 30 min. The extent of A2 fragment cleavage at each time point was determined by SDS-PAGE and western blotting and was expressed as percent of the A2 fragment cleaved. The rate of cleavage was expressed as the percent of A2 fragment cleaved per minute. In cases when the rates of cleavage were fast and became nonlinear with time, initial rates extrapolated to time 0 were used for comparison. In the SDS-PAGE and western blot analyses, the reaction mixtures were reduced and fractionated by SDS-PAGE on 4–20% gradient polyacrylamide gels (Bio-Rad Laboratories, Hercules, CA) after reduction. The fractionated products were transferred onto nitrocellulose membranes, blocked with 1% bovine serum albumin in Tris-buffered saline with 0.1% Tween-20 TBST (50 mM Tris HCl, pH 7.5, 150 mM NaCl, 0.1% Tween 20) for 30 minutes at room temperature, and probed with streptavidin-HRP conjugate (Thermal Scientific, Rockford, IL) diluted 1:10,000 in TBST containing 1% albumin. The nitrocellulose membranes were washed for 15 min in three changes of TBST and incubated with the chemiluminescent HRP substrate Immobilon Western HRP substrate peroxide solution (Millipore, Billerica, MA). The intensity of chemiluminescence was captured on an ImageQuant 350 imaging system (GE Healthcare, Piscataway, NJ). The extent of cleavage at each time point was expressed as percent of the VWF fragment cleaved.

## Supporting Information

Figure S1
**Time series of the C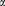 RMSD for helix 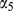 from the initial conformation for the wild-type and the three mutants.** Prior to calculating the RMSD, helix 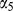 of each snapshot was aligned onto its conformation in the initial structure.(TIF)

Figure S2
**Time series of the C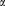 RMSD for helix 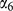 from the initial conformation for the wild-type and the three mutants.** Prior to calculating the RMSD, helix 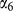 of each snapshot was aligned onto its conformation in the initial structure.(TIF)

Figure S3
**Time series of quantities measured during the simulation WT_pull_2 with the wild-type.** (**a**) Applied tensile force. Events observed during the simulations corresponding to force peaks (i.e., sharp increases followed by drops) are indicated. (**b**) Formation of secondary structure elements. The colors are explained in the legend on the right. The position of the Tyr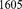-Met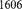 cleavage site is indicated by a red line and labeled on the right. (**c**) C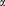 RMSD of the two C-terminus proximal helices 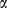
5 and 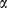
6. (**d**) Solvent accessible surface area of the Tyr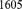-Met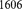 cleavage site. (**e**) C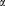 RMSD from the native state for the N-terminal part of the (residues 1497 to 1605) and the C-terminal part of the protein (residues 1606 to 1668). (**f**) Solvent accessible surface area of the minimum docking unit for ADAMTS13 (residues 1645–1668) identified in a previous experimental study [28].(TIF)

Figure S4
**Time series of quantities measured during the simulation WT_pull_2 with the wild-type.** (**a**) Applied tensile force. Events observed during the simulations corresponding to force peaks (i.e., sharp increases followed by drops) are indicated. (**b**) Formation of secondary structure elements. The colors are explained in the legend on the right. The position of the Tyr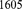-Met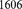 cleavage site is indicated by a red line and labeled on the right. (**c**) C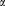 RMSD of the two C-terminus proximal helices 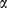
5 and 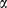
6. (**d**) Solvent accessible surface area of the Tyr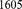-Met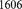 cleavage site. (**e**) C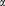 RMSD from the native state for the N-terminal part of the (residues 1497 to 1605) and the C-terminal part of the protein (residues 1606 to 1668). (**f**) Solvent accessible surface area of the minimum docking unit for ADAMTS13 (residues 1645–1668) identified in a previous experimental study [28].(TIF)

Figure S5
**Time series of quantities measured during the simulation WT_pull_3 with the wild-type.** (**a**) Applied tensile force. Events observed during the simulations corresponding to force peaks (i.e., sharp increases followed by drops) are indicated. (**b**) Formation of secondary structure elements. The colors are explained in the legend on the right. The position of the Tyr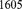-Met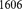 cleavage site is indicated by a red line and labeled on the right. (**c**) C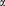 RMSD of the two C-terminus proximal helices 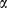
5 and 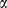
6. (**d**) Solvent accessible surface area of the Tyr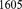-Met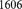 cleavage site. (**e**) C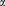 RMSD from the native state for the N-terminal part of the (residues 1497 to 1605) and the C-terminal part of the protein (residues 1606 to 1668). (**f**) Solvent accessible surface area of the minimum docking unit for ADAMTS13 (residues 1645–1668) identified in a previous experimental study [28].(TIF)

Figure S6
**Time series of quantities measured during the simulation L1657I_pull_1 with the wild-type.** (**a**) Applied tensile force. Events observed during the simulations corresponding to force peaks (i.e., sharp increases followed by drops) are indicated. (**b**) Formation of secondary structure elements. The colors are explained in the legend on the right. The position of the Tyr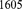-Met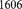 cleavage site is indicated by a red line and labeled on the right. (**c**) C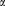 RMSD of the two C-terminus proximal helices 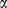
5 and 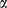
6. (**d**) Solvent accessible surface area of the Tyr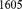-Met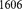 cleavage site. (**e**) C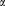 RMSD from the native state for the N-terminal part of the (residues 1497 to 1605) and the C-terminal part of the protein (residues 1606 to 1668). (**f**) Solvent accessible surface area of the minimum docking unit for ADAMTS13 (residues 1645–1668) identified in a previous experimental study [28].(TIF)

Figure S7
**Time series of quantities measured during the simulation L1657I_pull_2 with the wild-type.** (**a**) Applied tensile force. Events observed during the simulations corresponding to force peaks (i.e., sharp increases followed by drops) are indicated. (**b**) Formation of secondary structure elements. The colors are explained in the legend on the right. The position of the Tyr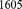-Met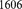 cleavage site is indicated by a red line and labeled on the right. (**c**) C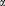 RMSD of the two C-terminus proximal helices 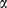
5 and 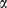
6. (**d**) Solvent accessible surface area of the Tyr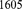-Met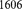 cleavage site. (**e**) C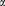 RMSD from the native state for the N-terminal part of the (residues 1497 to 1605) and the C-terminal part of the protein (residues 1606 to 1668). (**f**) Solvent accessible surface area of the minimum docking unit for ADAMTS13 (residues 1645–1668) identified in a previous experimental study [28].(TIF)

Figure S8
**Time series of quantities measured during the simulation L1657I_pull_3 with the wild-type.** (**a**) Applied tensile force. Events observed during the simulations corresponding to force peaks (i.e., sharp increases followed by drops) are indicated. (**b**) Formation of secondary structure elements. The colors are explained in the legend on the right. The position of the Tyr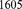-Met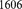 cleavage site is indicated by a red line and labeled on the right. (**c**) C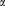 RMSD of the two C-terminus proximal helices 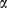
5 and 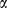
6. (**d**) Solvent accessible surface area of the Tyr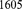-Met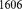 cleavage site. (**e**) C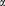 RMSD from the native state for the N-terminal part of the (residues 1497 to 1605) and the C-terminal part of the protein (residues 1606 to 1668). (**f**) Solvent accessible surface area of the minimum docking unit for ADAMTS13 (residues 1645–1668) identified in a previous experimental study [28].(TIF)

Figure S9
**Time series of quantities measured during the simulation I1628T_pull_1 with the wild-type.** (**a**) Applied tensile force. Events observed during the simulations corresponding to force peaks (i.e., sharp increases followed by drops) are indicated. (**b**) Formation of secondary structure elements. The colors are explained in the legend on the right. The position of the Tyr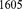-Met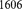 cleavage site is indicated by a red line and labeled on the right. (**c**) C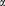 RMSD of the two C-terminus proximal helices 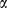
5 and 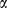
6. (**d**) Solvent accessible surface area of the Tyr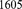-Met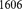 cleavage site. (**e**) C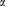 RMSD from the native state for the N-terminal part of the (residues 1497 to 1605) and the C-terminal part of the protein (residues 1606 to 1668). (**f**) Solvent accessible surface area of the minimum docking unit for ADAMTS13 (residues 1645–1668) identified in a previous experimental study [28].(TIF)

Figure S10
**Time series of quantities measured during the simulation I1628T_pull_2 with the wild-type.** (**a**) Applied tensile force. Events observed during the simulations corresponding to force peaks (i.e., sharp increases followed by drops) are indicated. (**b**) Formation of secondary structure elements. The colors are explained in the legend on the right. The position of the Tyr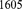-Met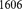 cleavage site is indicated by a red line and labeled on the right. (**c**) C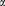 RMSD of the two C-terminus proximal helices 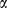
5 and 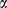
6. (**d**) Solvent accessible surface area of the Tyr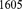-Met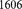 cleavage site. (**e**) C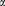 RMSD from the native state for the N-terminal part of the (residues 1497 to 1605) and the C-terminal part of the protein (residues 1606 to 1668). (**f**) Solvent accessible surface area of the minimum docking unit for ADAMTS13 (residues 1645–1668) identified in a previous experimental study [28].(TIF)

Figure S11
**Time series of quantities measured during the simulation I1628T_pull_3 with the wild-type.** (**a**) Applied tensile force. Events observed during the simulations corresponding to force peaks (i.e., sharp increases followed by drops) are indicated. (**b**) Formation of secondary structure elements. The colors are explained in the legend on the right. The position of the Tyr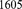-Met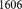 cleavage site is indicated by a red line and labeled on the right. (**c**) C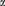 RMSD of the two C-terminus proximal helices 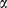
5 and 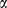
6. (**d**) Solvent accessible surface area of the Tyr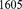-Met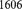 cleavage site. (**e**) C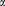 RMSD from the native state for the N-terminal part of the (residues 1497 to 1605) and the C-terminal part of the protein (residues 1606 to 1668). (**f**) Solvent accessible surface area of the minimum docking unit for ADAMTS13 (residues 1645–1668) identified in a previous experimental study [28].(TIF)

Figure S12
**Time series of quantities measured during the simulation E1638K_pull_1 with the wild-type.** (**a**) Applied tensile force. Events observed during the simulations corresponding to force peaks (i.e., sharp increases followed by drops) are indicated. (**b**) Formation of secondary structure elements. The colors are explained in the legend on the right. The position of the Tyr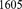-Met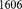 cleavage site is indicated by a red line and labeled on the right. (**c**) C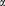 RMSD of the two C-terminus proximal helices 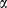
5 and 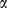
6. (**d**) Solvent accessible surface area of the Tyr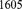-Met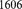 cleavage site. (**e**) C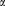 RMSD from the native state for the N-terminal part of the (residues 1497 to 1605) and the C-terminal part of the protein (residues 1606 to 1668). (**f**) Solvent accessible surface area of the minimum docking unit for ADAMTS13 (residues 1645–1668) identified in a previous experimental study [28].(TIF)

Figure S13
**Time series of quantities measured during the simulation E1638K_pull_2 with the wild-type.** (**a**) Applied tensile force. Events observed during the simulations corresponding to force peaks (i.e., sharp increases followed by drops) are indicated. (**b**) Formation of secondary structure elements. The colors are explained in the legend on the right. The position of the Tyr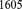-Met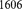 cleavage site is indicated by a red line and labeled on the right. (**c**) C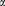 RMSD of the two C-terminus proximal helices 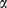
5 and 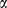
6. (**d**) Solvent accessible surface area of the Tyr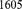-Met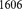 cleavage site. (**e**) C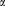 RMSD from the native state for the N-terminal part of the (residues 1497 to 1605) and the C-terminal part of the protein (residues 1606 to 1668). (**f**) Solvent accessible surface area of the minimum docking unit for ADAMTS13 (residues 1645–1668) identified in a previous experimental study [28].(TIF)

Figure S14
**Time series of quantities measured during the simulation E1638K_pull_3 with the wild-type.** (**a**) Applied tensile force. Events observed during the simulations corresponding to force peaks (i.e., sharp increases followed by drops) are indicated. (**b**) Formation of secondary structure elements. The colors are explained in the legend on the right. The position of the Tyr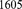-Met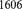 cleavage site is indicated by a red line and labeled on the right. (**c**) C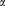 RMSD of the two C-terminus proximal helices 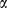
5 and 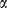
6. (**d**) Solvent accessible surface area of the Tyr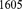-Met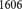 cleavage site. (**e**) C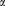 RMSD from the native state for the N-terminal part of the (residues 1497 to 1605) and the C-terminal part of the protein (residues 1606 to 1668). (**f**) Solvent accessible surface area of the minimum docking unit for ADAMTS13 (residues 1645–1668) identified in a previous experimental study [28].(TIF)

Figure S15
**Time series of the force and SASA of the C-terminal hydrophobic core.** The force peak was determined by identifying the time point when the SASA exceeds 50 Å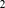 and searching for the highest value of the force within a 400 ps time window. 20-ps running average is indicated in red for the force and in blue for the SASA, respectively. (**a**) Wild-type. (**b**) I1628T. (**c**) L1657I. (**d**) E1638K.(TIF)

Figure S16
**Time series of the force and SASA of the C-terminal hydrophobic core.** The force peak was determined by identifying the time point when the SASA exceeds 50 Å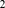 and searching for the highest value of the force within a 400 ps time window. 20-ps running average is indicated in red for the force and in blue for the SASA, respectively. (**a**) Wild-type. (**b**) I1628T. (**c**) L1657I. (**d**) E1638K.(TIF)

Movie S1 **Movie showing the unfolding of the A2 domain under tensile force in the simulation WT_pull_1.** Side chains of residues located in the C-terminal hydrophobic core, in the cleavage site and of the cysteine residues in the C-terminus are shown in the stick and ball representation. The backbone of the cleavage site is colored in red. This movie was generated with the program VMD [49].
(MPG)

Movie S2 **Movie showing the unfolding of the A2 domain under tensile force in the simulation WT_pull_1 zooming in the core of the protein.** Side chains of residues located in the C-terminal hydrophobic core, in the cleavage site and of the cysteine residues in the C-terminus are shown in the stick and ball representation. The backbone of the cleavage site is colored in red. This movie was generated with the program VMD [49].
(MPG)
