# Supplementary figures and images for: Structural Basis of Type 2A von Willebrand Disease Investigated by Molecular Dynamics Simulations and Experiments
Source: PLoS One. 2012 Oct 23;7(10):e45207. doi: 10.1371/journal.pone.0045207 (PMC3479114; doi:10.1371/journal.pone.0045207)

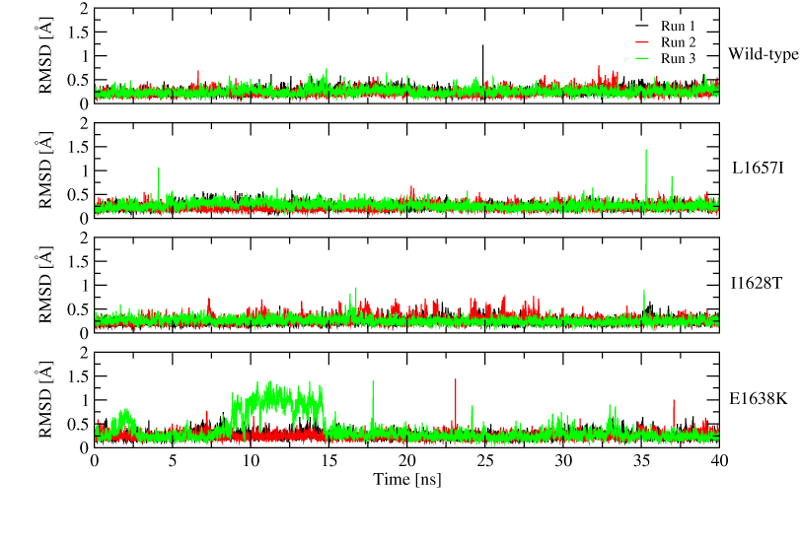

Supplement: Figure S1 — Time series of the C RMSD for helix from the initial conformation for the wild-type and the three mutants. Prior to calculating the RMSD, helix of each snapshot was aligned onto its conformation in the initial structure. (TIF) [file pone.0045207.s001.tif]

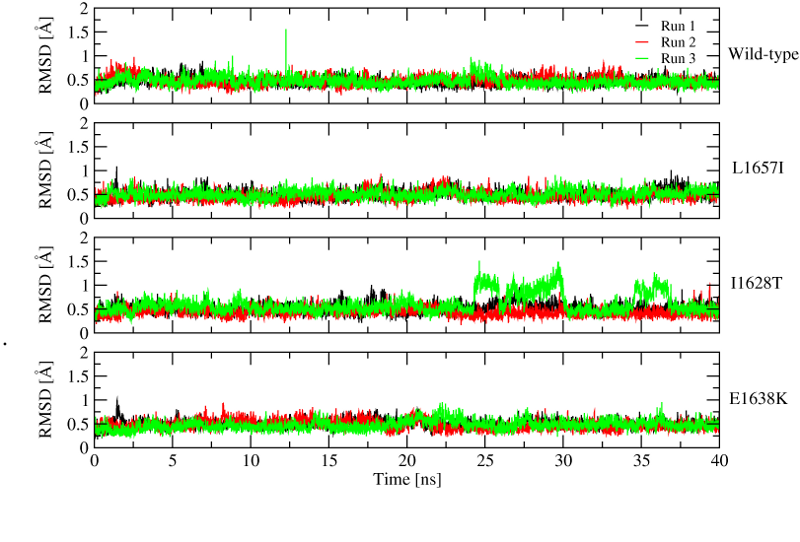

Supplement: Figure S2 — Time series of the C RMSD for helix from the initial conformation for the wild-type and the three mutants. Prior to calculating the RMSD, helix of each snapshot was aligned onto its conformation in the initial structure. (TIF) [file pone.0045207.s002.tif]

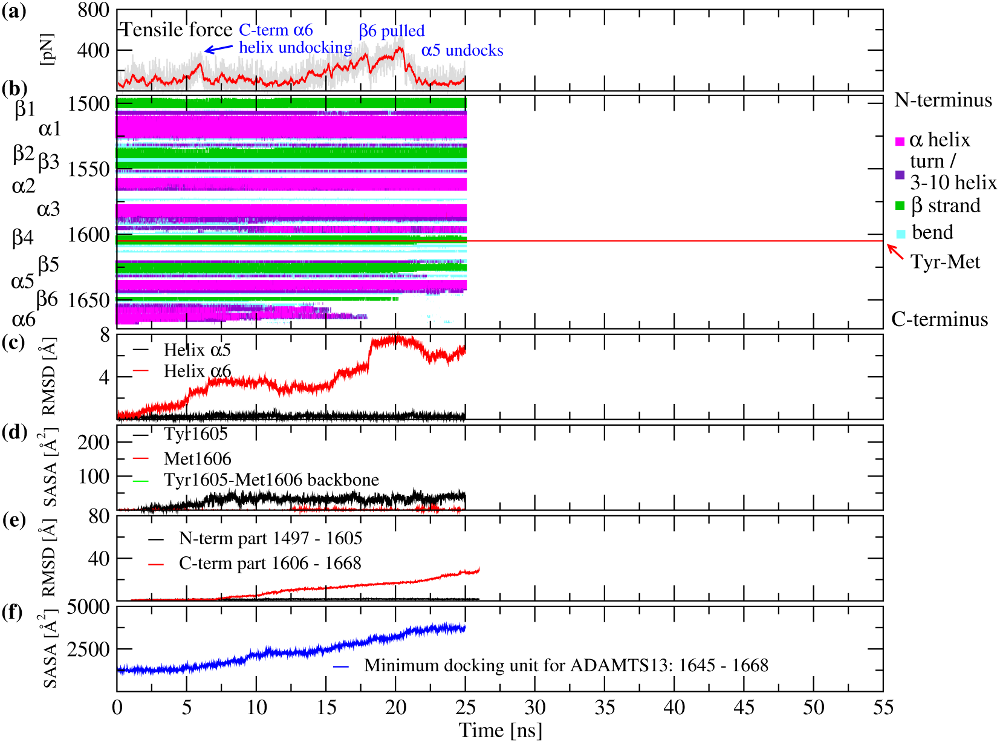

Supplement: Figure S3 — Time series of quantities measured during the simulation WT_pull_2 with the wild-type. (a) Applied tensile force. Events observed during the simulations corresponding to force peaks (i.e., sharp increases followed by drops) are indicated. (b) Formation of secondary structure elements. The colors are explained in the legend on the right. The position of the Tyr-Met cleavage site is indicated by a red line and labeled on the right. (c) C RMSD of the two C-terminus proximal helices 5 and 6. (d) Solvent accessible surface area of the Tyr-Met cleavage site. (e) C RMSD from the native state for the N-terminal part of the (residues 1497 to 1605) and the C-terminal part of the protein (residues 1606 to 1668). (f) Solvent accessible surface area of the minimum docking unit for ADAMTS13 (residues 1645–1668) identified in a previous experimental study [28]. (TIF) [file pone.0045207.s003.tif]

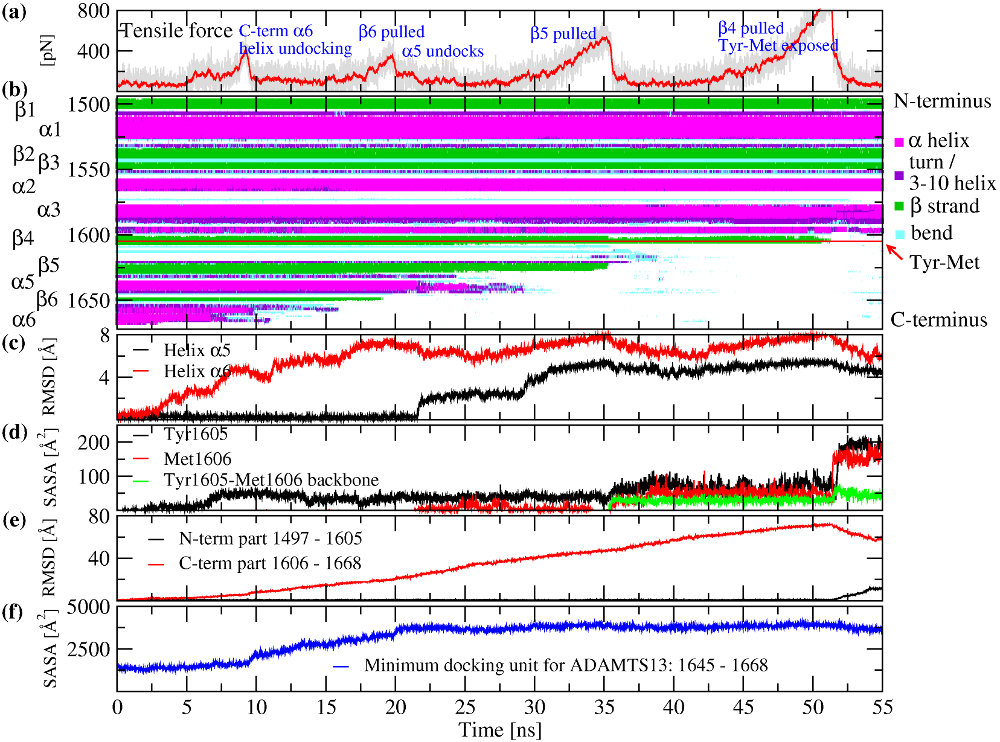

Supplement: Figure S4 — Time series of quantities measured during the simulation WT_pull_2 with the wild-type. (a) Applied tensile force. Events observed during the simulations corresponding to force peaks (i.e., sharp increases followed by drops) are indicated. (b) Formation of secondary structure elements. The colors are explained in the legend on the right. The position of the Tyr-Met cleavage site is indicated by a red line and labeled on the right. (c) C RMSD of the two C-terminus proximal helices 5 and 6. (d) Solvent accessible surface area of the Tyr-Met cleavage site. (e) C RMSD from the native state for the N-terminal part of the (residues 1497 to 1605) and the C-terminal part of the protein (residues 1606 to 1668). (f) Solvent accessible surface area of the minimum docking unit for ADAMTS13 (residues 1645–1668) identified in a previous experimental study [28]. (TIF) [file pone.0045207.s004.tif]

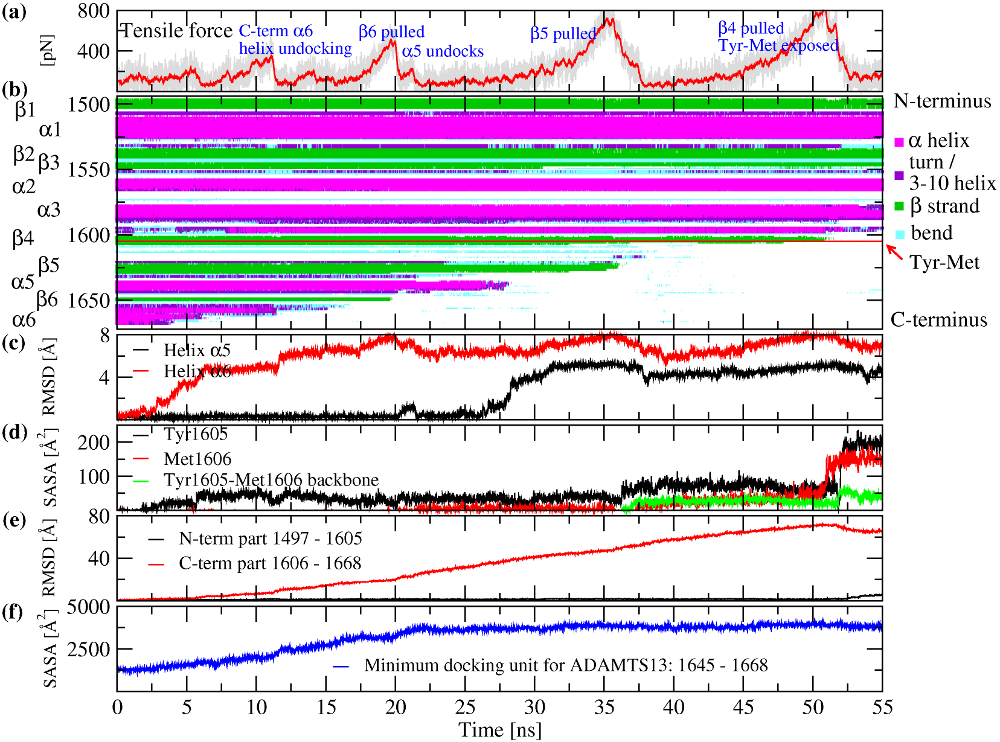

Supplement: Figure S5 — Time series of quantities measured during the simulation WT_pull_3 with the wild-type. (a) Applied tensile force. Events observed during the simulations corresponding to force peaks (i.e., sharp increases followed by drops) are indicated. (b) Formation of secondary structure elements. The colors are explained in the legend on the right. The position of the Tyr-Met cleavage site is indicated by a red line and labeled on the right. (c) C RMSD of the two C-terminus proximal helices 5 and 6. (d) Solvent accessible surface area of the Tyr-Met cleavage site. (e) C RMSD from the native state for the N-terminal part of the (residues 1497 to 1605) and the C-terminal part of the protein (residues 1606 to 1668). (f) Solvent accessible surface area of the minimum docking unit for ADAMTS13 (residues 1645–1668) identified in a previous experimental study [28]. (TIF) [file pone.0045207.s005.tif]

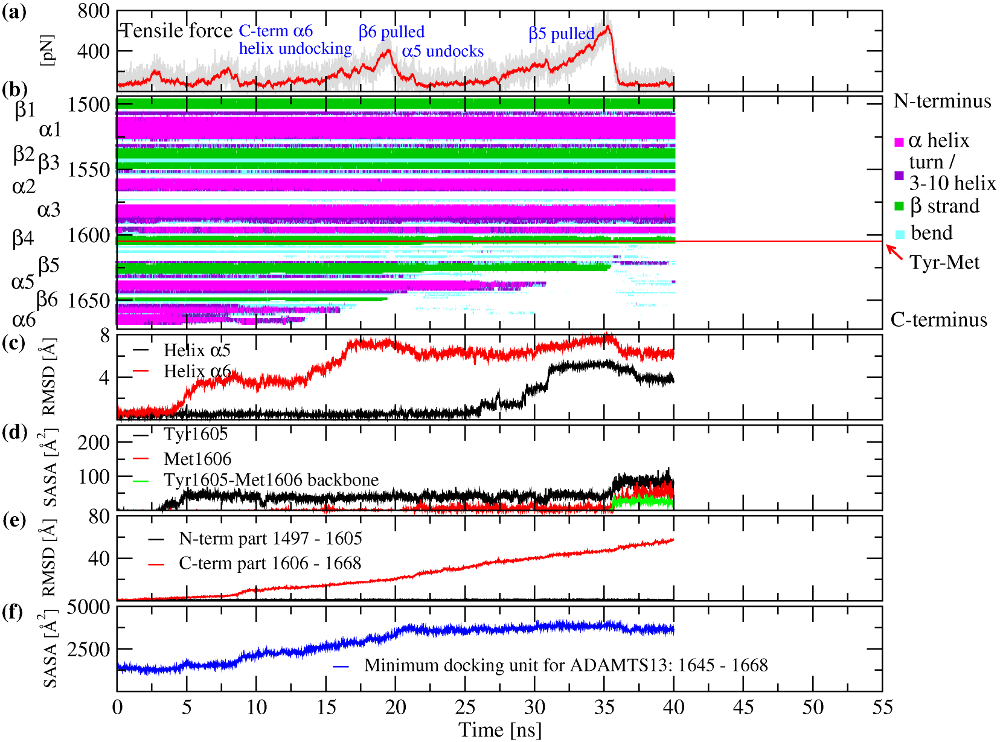

Supplement: Figure S6 — Time series of quantities measured during the simulation L1657I_pull_1 with the wild-type. (a) Applied tensile force. Events observed during the simulations corresponding to force peaks (i.e., sharp increases followed by drops) are indicated. (b) Formation of secondary structure elements. The colors are explained in the legend on the right. The position of the Tyr-Met cleavage site is indicated by a red line and labeled on the right. (c) C RMSD of the two C-terminus proximal helices 5 and 6. (d) Solvent accessible surface area of the Tyr-Met cleavage site. (e) C RMSD from the native state for the N-terminal part of the (residues 1497 to 1605) and the C-terminal part of the protein (residues 1606 to 1668). (f) Solvent accessible surface area of the minimum docking unit for ADAMTS13 (residues 1645–1668) identified in a previous experimental study [28]. (TIF) [file pone.0045207.s006.tif]

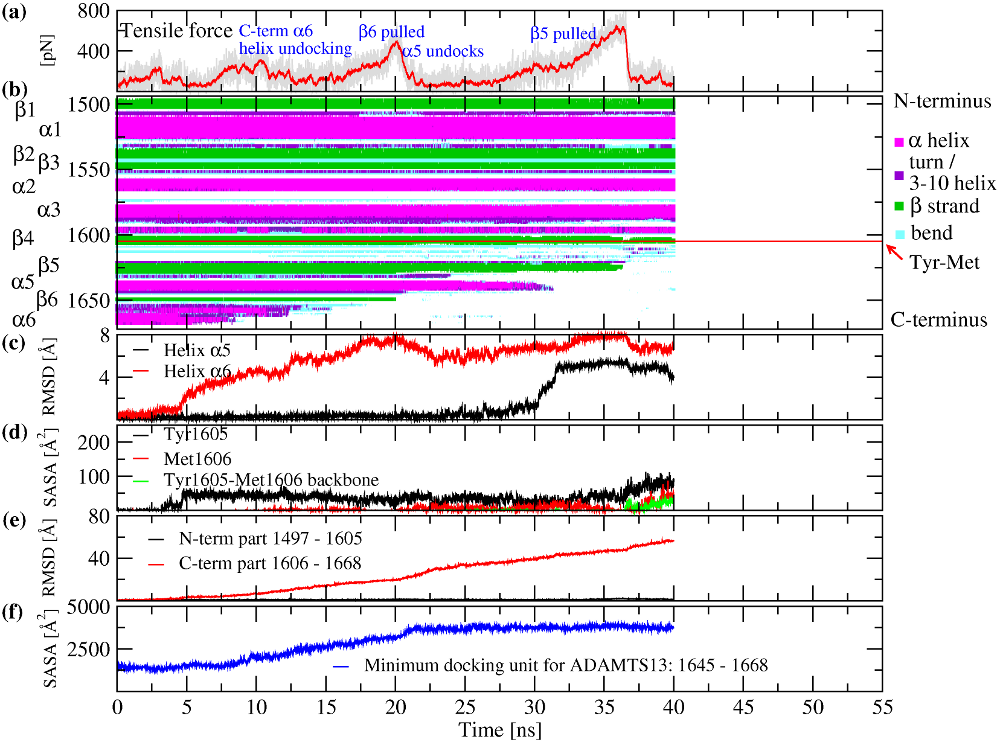

Supplement: Figure S7 — Time series of quantities measured during the simulation L1657I_pull_2 with the wild-type. (a) Applied tensile force. Events observed during the simulations corresponding to force peaks (i.e., sharp increases followed by drops) are indicated. (b) Formation of secondary structure elements. The colors are explained in the legend on the right. The position of the Tyr-Met cleavage site is indicated by a red line and labeled on the right. (c) C RMSD of the two C-terminus proximal helices 5 and 6. (d) Solvent accessible surface area of the Tyr-Met cleavage site. (e) C RMSD from the native state for the N-terminal part of the (residues 1497 to 1605) and the C-terminal part of the protein (residues 1606 to 1668). (f) Solvent accessible surface area of the minimum docking unit for ADAMTS13 (residues 1645–1668) identified in a previous experimental study [28]. (TIF) [file pone.0045207.s007.tif]

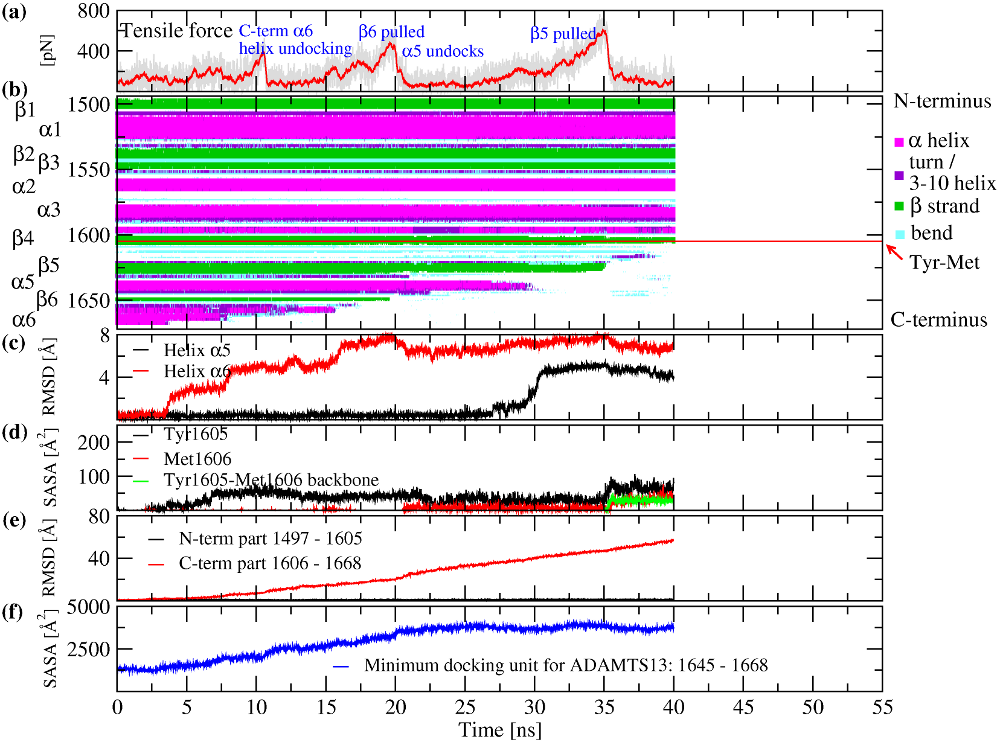

Supplement: Figure S8 — Time series of quantities measured during the simulation L1657I_pull_3 with the wild-type. (a) Applied tensile force. Events observed during the simulations corresponding to force peaks (i.e., sharp increases followed by drops) are indicated. (b) Formation of secondary structure elements. The colors are explained in the legend on the right. The position of the Tyr-Met cleavage site is indicated by a red line and labeled on the right. (c) C RMSD of the two C-terminus proximal helices 5 and 6. (d) Solvent accessible surface area of the Tyr-Met cleavage site. (e) C RMSD from the native state for the N-terminal part of the (residues 1497 to 1605) and the C-terminal part of the protein (residues 1606 to 1668). (f) Solvent accessible surface area of the minimum docking unit for ADAMTS13 (residues 1645–1668) identified in a previous experimental study [28]. (TIF) [file pone.0045207.s008.tif]

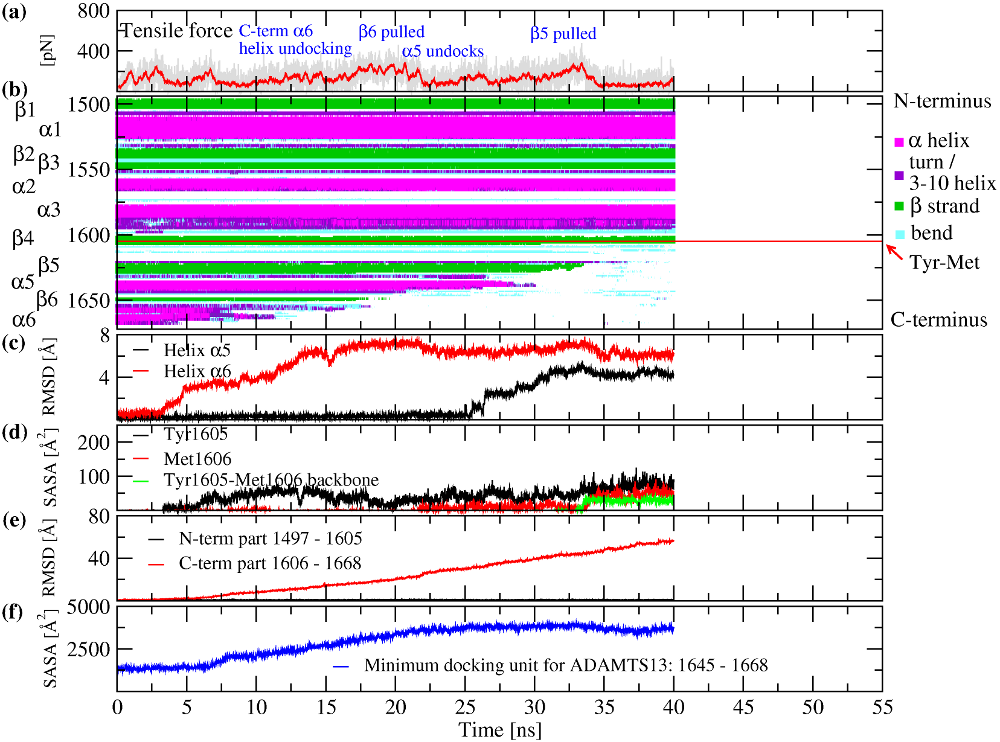

Supplement: Figure S9 — Time series of quantities measured during the simulation I1628T_pull_1 with the wild-type. (a) Applied tensile force. Events observed during the simulations corresponding to force peaks (i.e., sharp increases followed by drops) are indicated. (b) Formation of secondary structure elements. The colors are explained in the legend on the right. The position of the Tyr-Met cleavage site is indicated by a red line and labeled on the right. (c) C RMSD of the two C-terminus proximal helices 5 and 6. (d) Solvent accessible surface area of the Tyr-Met cleavage site. (e) C RMSD from the native state for the N-terminal part of the (residues 1497 to 1605) and the C-terminal part of the protein (residues 1606 to 1668). (f) Solvent accessible surface area of the minimum docking unit for ADAMTS13 (residues 1645–1668) identified in a previous experimental study [28]. (TIF) [file pone.0045207.s009.tif]

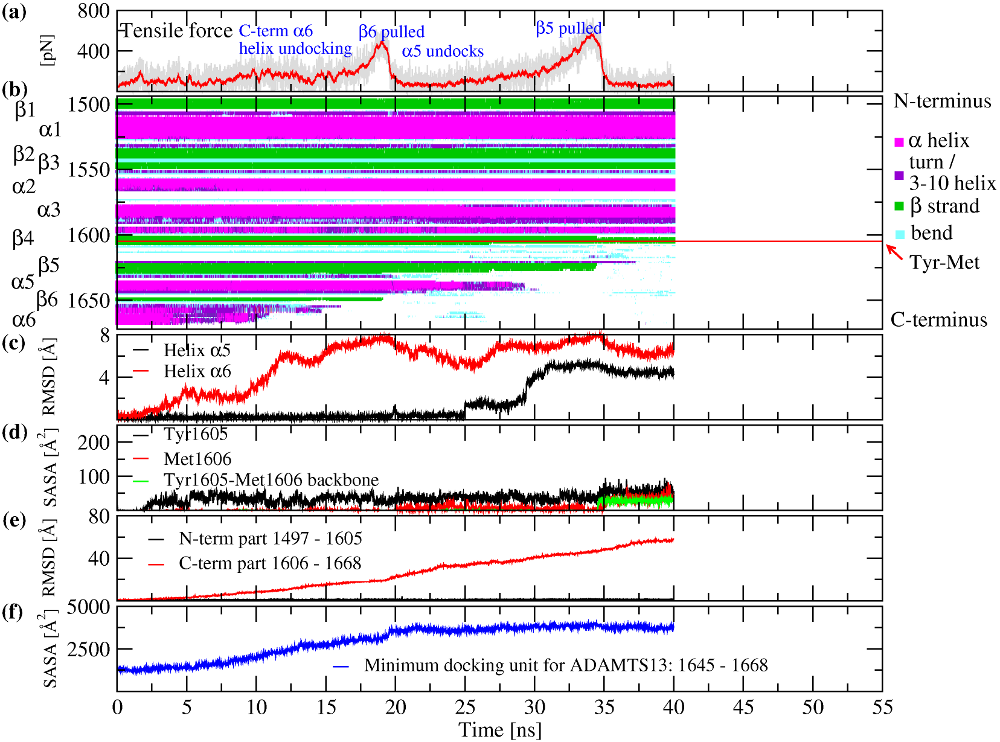

Supplement: Figure S10 — Time series of quantities measured during the simulation I1628T_pull_2 with the wild-type. (a) Applied tensile force. Events observed during the simulations corresponding to force peaks (i.e., sharp increases followed by drops) are indicated. (b) Formation of secondary structure elements. The colors are explained in the legend on the right. The position of the Tyr-Met cleavage site is indicated by a red line and labeled on the right. (c) C RMSD of the two C-terminus proximal helices 5 and 6. (d) Solvent accessible surface area of the Tyr-Met cleavage site. (e) C RMSD from the native state for the N-terminal part of the (residues 1497 to 1605) and the C-terminal part of the protein (residues 1606 to 1668). (f) Solvent accessible surface area of the minimum docking unit for ADAMTS13 (residues 1645–1668) identified in a previous experimental study [28]. (TIF) [file pone.0045207.s010.tif]

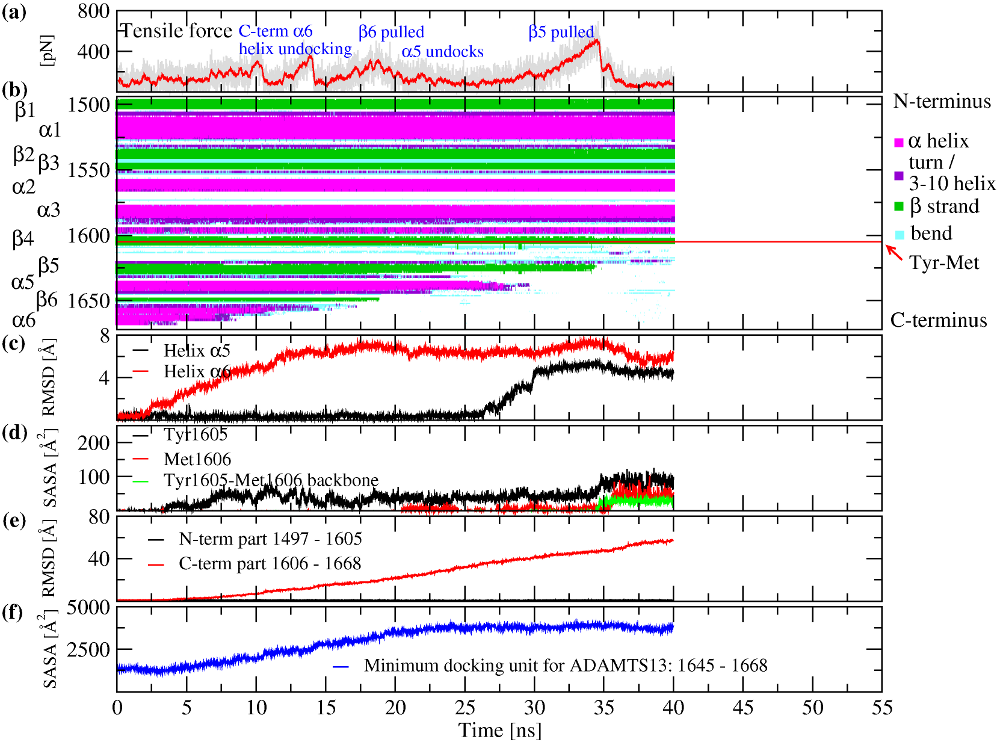

Supplement: Figure S11 — Time series of quantities measured during the simulation I1628T_pull_3 with the wild-type. (a) Applied tensile force. Events observed during the simulations corresponding to force peaks (i.e., sharp increases followed by drops) are indicated. (b) Formation of secondary structure elements. The colors are explained in the legend on the right. The position of the Tyr-Met cleavage site is indicated by a red line and labeled on the right. (c) C RMSD of the two C-terminus proximal helices 5 and 6. (d) Solvent accessible surface area of the Tyr-Met cleavage site. (e) C RMSD from the native state for the N-terminal part of the (residues 1497 to 1605) and the C-terminal part of the protein (residues 1606 to 1668). (f) Solvent accessible surface area of the minimum docking unit for ADAMTS13 (residues 1645–1668) identified in a previous experimental study [28]. (TIF) [file pone.0045207.s011.tif]

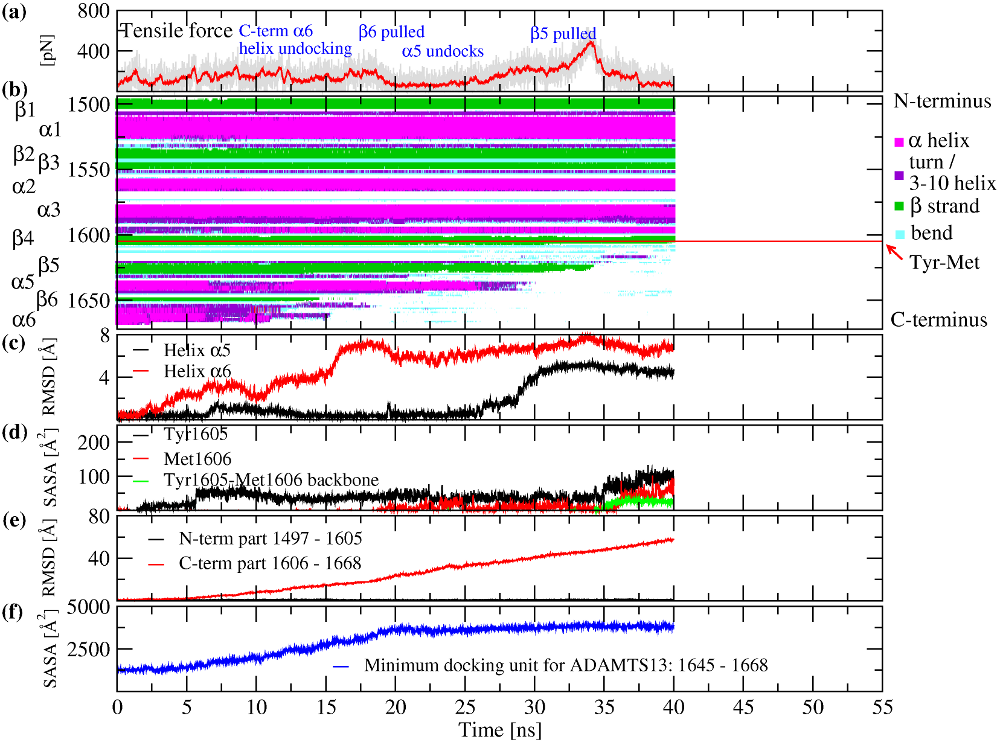

Supplement: Figure S12 — Time series of quantities measured during the simulation E1638K_pull_1 with the wild-type. (a) Applied tensile force. Events observed during the simulations corresponding to force peaks (i.e., sharp increases followed by drops) are indicated. (b) Formation of secondary structure elements. The colors are explained in the legend on the right. The position of the Tyr-Met cleavage site is indicated by a red line and labeled on the right. (c) C RMSD of the two C-terminus proximal helices 5 and 6. (d) Solvent accessible surface area of the Tyr-Met cleavage site. (e) C RMSD from the native state for the N-terminal part of the (residues 1497 to 1605) and the C-terminal part of the protein (residues 1606 to 1668). (f) Solvent accessible surface area of the minimum docking unit for ADAMTS13 (residues 1645–1668) identified in a previous experimental study [28]. (TIF) [file pone.0045207.s012.tif]

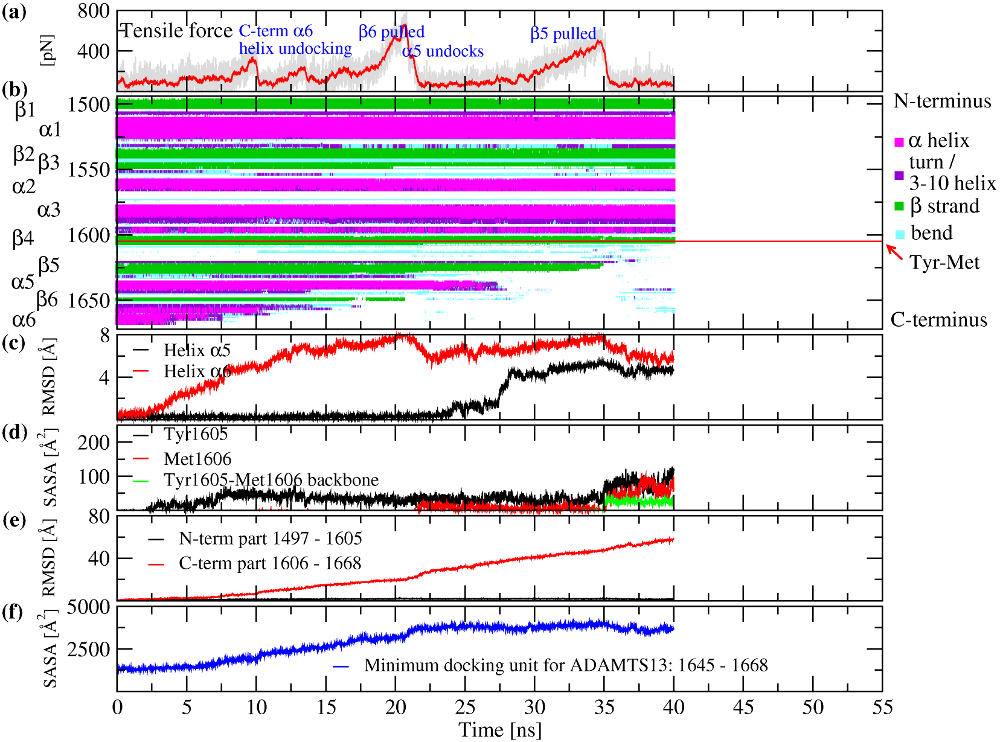

Supplement: Figure S13 — Time series of quantities measured during the simulation E1638K_pull_2 with the wild-type. (a) Applied tensile force. Events observed during the simulations corresponding to force peaks (i.e., sharp increases followed by drops) are indicated. (b) Formation of secondary structure elements. The colors are explained in the legend on the right. The position of the Tyr-Met cleavage site is indicated by a red line and labeled on the right. (c) C RMSD of the two C-terminus proximal helices 5 and 6. (d) Solvent accessible surface area of the Tyr-Met cleavage site. (e) C RMSD from the native state for the N-terminal part of the (residues 1497 to 1605) and the C-terminal part of the protein (residues 1606 to 1668). (f) Solvent accessible surface area of the minimum docking unit for ADAMTS13 (residues 1645–1668) identified in a previous experimental study [28]. (TIF) [file pone.0045207.s013.tif]

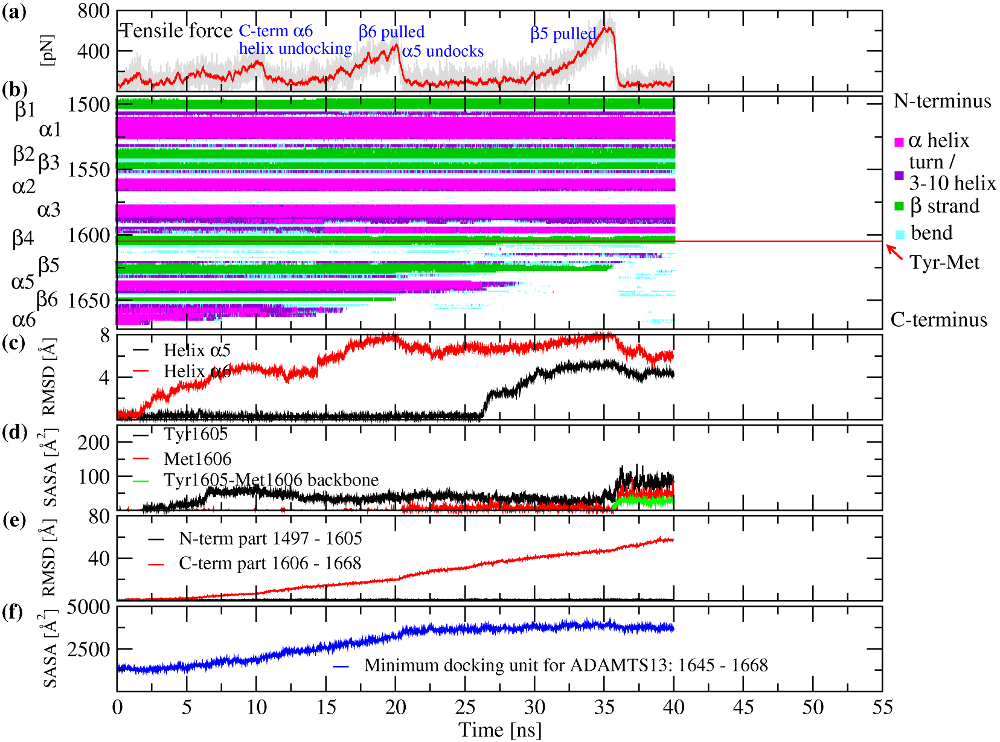

Supplement: Figure S14 — Time series of quantities measured during the simulation E1638K_pull_3 with the wild-type. (a) Applied tensile force. Events observed during the simulations corresponding to force peaks (i.e., sharp increases followed by drops) are indicated. (b) Formation of secondary structure elements. The colors are explained in the legend on the right. The position of the Tyr-Met cleavage site is indicated by a red line and labeled on the right. (c) C RMSD of the two C-terminus proximal helices 5 and 6. (d) Solvent accessible surface area of the Tyr-Met cleavage site. (e) C RMSD from the native state for the N-terminal part of the (residues 1497 to 1605) and the C-terminal part of the protein (residues 1606 to 1668). (f) Solvent accessible surface area of the minimum docking unit for ADAMTS13 (residues 1645–1668) identified in a previous experimental study [28]. (TIF) [file pone.0045207.s014.tif]

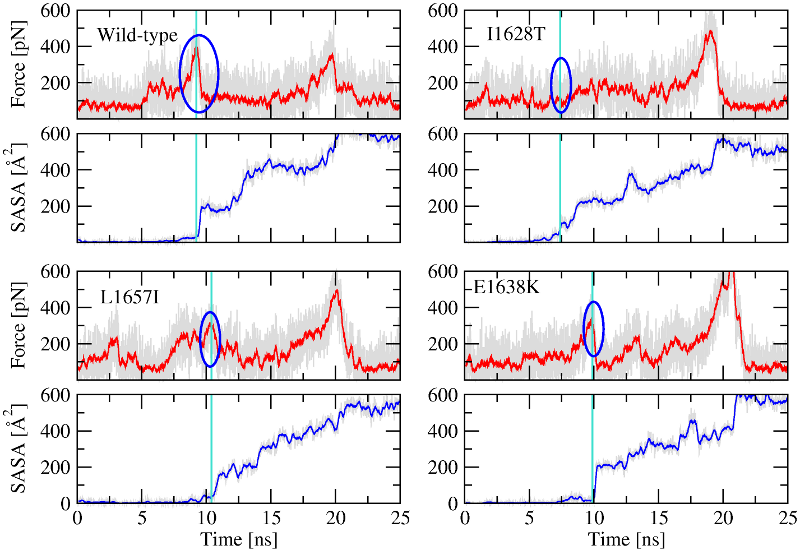

Supplement: Figure S15 — Time series of the force and SASA of the C-terminal hydrophobic core. The force peak was determined by identifying the time point when the SASA exceeds 50 Å and searching for the highest value of the force within a 400 ps time window. 20-ps running average is indicated in red for the force and in blue for the SASA, respectively. (a) Wild-type. (b) I1628T. (c) L1657I. (d) E1638K. (TIF) [file pone.0045207.s015.tif]

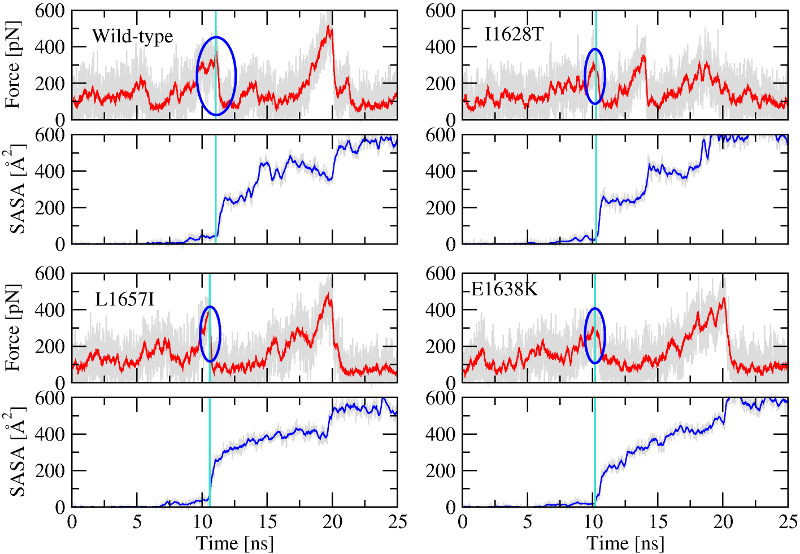

Supplement: Figure S16 — Time series of the force and SASA of the C-terminal hydrophobic core. The force peak was determined by identifying the time point when the SASA exceeds 50 Å and searching for the highest value of the force within a 400 ps time window. 20-ps running average is indicated in red for the force and in blue for the SASA, respectively. (a) Wild-type. (b) I1628T. (c) L1657I. (d) E1638K. (TIF) [file pone.0045207.s016.tif]
